# Local active memristive oscillator enables controllable complex behaviours and frequency domain extraction

**DOI:** 10.1093/nsr/nwaf546

**Published:** 2025-12-08

**Authors:** Yanghao Wang, Pek Jun Tiw, Yuheng Liu, Yaoyu Tao, Teng Zhang, Yuchao Yang

**Affiliations:** Beijing Advanced Innovation Center for Integrated Circuits, School of Integrated Circuits, Peking University, Beijing 100871, China; Beijing Advanced Innovation Center for Integrated Circuits, School of Integrated Circuits, Peking University, Beijing 100871, China; Beijing Advanced Innovation Center for Integrated Circuits, School of Integrated Circuits, Peking University, Beijing 100871, China; Institute of Artificial Intelligence, Peking University, Beijing 100871, China; Beijing Advanced Innovation Center for Integrated Circuits, School of Integrated Circuits, Peking University, Beijing 100871, China; State Key Laboratory of Multimedia Information Processing, School of Computer Science, Peking University, Beijing 100871, China; Beijing Advanced Innovation Center for Integrated Circuits, School of Integrated Circuits, Peking University, Beijing 100871, China; Guangdong Provincial Key Laboratory of In-Memory Computing Chips, School of Electronic and Computer Engineering, Peking University, Shenzhen 518055, China; Institute of Artificial Intelligence, Peking University, Beijing 100871, China; Center for Brain Inspired Intelligence, Chinese Institute for Brain Research (CIBR), Beijing 102206, China

**Keywords:** VO_2_ oscillators, non-linear dynamics, local activity, injection-based control, edge of chaos

## Abstract

Physical non-linearities near the Mott transition exhibit substantial potential for neuromorphic computing. The complex computational behaviour stems from their intrinsic local active characteristics. Most studies focus on decay dynamics or regular oscillations, treating Mott devices primarily as simple threshold elements. Challenges remain in connecting measurable material properties to more complex device dynamics and their control methods through a unified theoretical model. Here, we develop a thermodynamic compact model for vanadium oxide devices based on electrical measurements and the local active principle. Utilizing the non-linearities near the Mott transition, we propose an injection-based control method to regulate behaviours of non-linear oscillators, such as frequency division, stochastic oscillations and frequency locking. Finally, a single device operating at the edge of chaos demonstrates exceptional capability in extracting information in the frequency domain within a physical computing framework, achieving performance equivalent to a two-layer convolutional neural network on the same task. This work facilitates a paradigm shift from traditional local passive devices to local active devices, bridging the physical non-linearities, circuit dynamics and computational theory to advance dynamic neuromorphic computing.

## Teaser

This work develops a thermodynamic model for VO_2_ devices, enabling injection-based control of nonlinear oscillators to achieve advanced neuromorphic computing capabilities at the edge of chaos.

## INTRODUCTION

The computational capacity of physical computing arises from the governing physical laws and inherent dynamics of nanodevices. For example, memristive crossbar arrays can perform vector–matrix multiplication (VMM) operations with high energy efficiency by exploiting Ohm’s law and Kirchhoff’s laws [1–4]. These chips employ non-volatile memristors whose dynamics are characterized by approximately linear conductance modulation and long-term plasticity [5,6]. On the other hand, volatile memristors, owing to their non-linear current–voltage (I–V) responses and short-term plasticity, can support reservoir computing [7–10]—an approach offering low training costs and promising efficient temporal signal processing. According to the memristor theory [11], both belong to the category of local passive memristors, as dissipative circuit elements suppress the growth of the fluctuations. On the contrary, memristors with local active characteristics can amplify fluctuations and exhibit complex behaviours [12], representing a novel paradigm for storing and processing more information within a single device [13].

**Figure 1.**
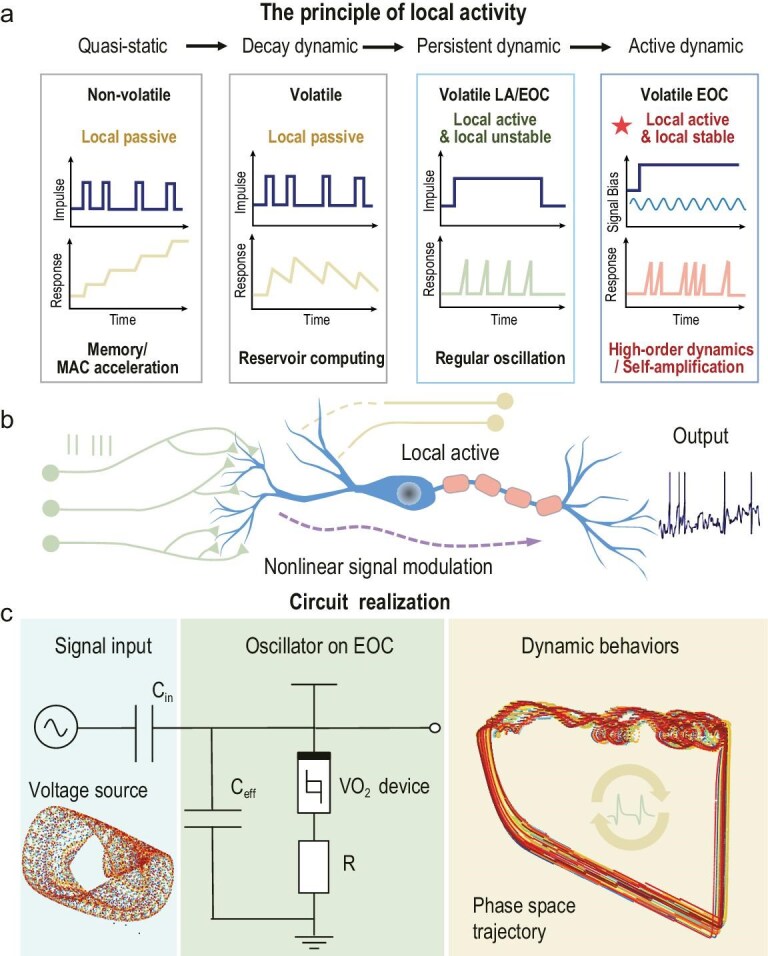
Physical neurons and oscillators working on the EOC for non-linear modulation. (a) Different memristors’ dynamics based on the principle of local activity from the quasi-static dynamic to the active dynamic, exhibiting characteristics of memory/MAC (multiply–accumulate operation), reservoir computing, regular oscillation, and high-order dynamics/self-amplification. (b) Local active biological neurons with intrinsic complexity realize non-linear signal modulation. (c) The circuit realization of injection control with VO_2_ devices and its dynamic behaviours. The oscillator’s dynamic behaviour exhibits a complex oscillatory loop structure in the phase plane.

Electrothermal Mott devices, such as those with vanadium oxide (VO_2_) or niobium dioxide (NbO_2_), possess pronounced physical non-linear dynamics [14]. In most previous studies, these devices were regarded as simple threshold-switching elements used to construct artificial neurons and regular oscillators [15–19]. However, they have recently been identified and experimentally validated as local active memristors [20–22]. When their local activity is considered and the operating bias is finely tuned, a single device can generate diverse complex action potentials [23], chaotic oscillations [24] and axon-like self-amplifying signal transmission [25]. The temperature-dependent non-linearities in electrical and thermal conductivities have been theoretically and experimentally established as the origin of local activity in Mott devices [20,21,26,27]. The theoretical foundation is rooted in the principle of local activity proposed by Chua [11,12,28]. It provides a unified criterion for the operation of biological/artificial neurons at the edge of chaos (EOC) when they exhibit both locally stable and locally active properties [28]. Here, local activity denotes the capability to amplify fluctuations, whereas local stability prevents unlimited growth of small perturbations. In contrast, local passive memristors used for VMM are designed to minimize drift and thermal fluctuations to maintain stable conductance states. Based on the local activity principle, issues such as the presence of negative elements in non-linear circuits, Hodgkin–Huxley neuron firing behaviours and Turing instabilities can be addressed in theoretical frameworks [12,29,30]. Accordingly, as illustrated in Fig. 1a, the local activity principle leads to a classification of memristor dynamics—from simple to complex—spanning locally passive to locally active behaviours, unveiling a new paradigm for physical computing at the EOC. A more detailed description of local active theory can be found in Note S1.

Currently, neurons in artificial neural networks are oversimplified to perform summation and non-linear activation, whereas a single biological neuron in the human brain can serve as a sophisticated information-processing unit [31]. Cortical neurons exhibit rich spike patterns and heterogeneous firing rates, such as periodic spiking, bursting and sub/super-threshold active dynamics [32,33]. When scaled to large and complex neuron networks, the additional action potentials contribute to the formation of high-order patterns in population information [34,35]. Perturbations in individual neurons can lead to behavioural consequences and may reshape sensory representations [36,37].

Membrane potential fluctuations driven by network dynamics can enable circuits to operate in distinct modes. Currently, most dynamic computing units are designed for specific task types, and the mechanisms governing transitions between functional modes remain poorly understood. In natural dynamic behaviours, hippocampal neurons rapidly switch their core computations to represent relevant behavioural variables, thereby supporting behavioural flexibility [38]. Moreover, cortical neurons exhibit irregular pulse patterns and heterogeneous firing rates over time. These features emerge in model circuits operating in wave-driven regimes [39], where membrane potential fluctuations are governed by network dynamics, as illustrated in Fig. 1b. Exploring the complexity of computational elements is crucial to support and control more complex dynamic behaviours [13].

In this work, we explore the controllable dynamic behaviours induced by small injection signals on a VO_2_ oscillator biased at the critical EOC region. First, we fabricated monocrystalline VO_2_ memristive devices with high uniformity, and developed a physical compact model grounded in the principle of local activity. We found that the critical EOC point exhibits robustness against ambient temperature fluctuations, as the current remains approximately constant over a certain temperature range. We then experimentally biased the VO_2_ oscillator at the critical EOC point and perturbed it with signals of varying frequencies. Owing to its non-linear physical dynamics, the VO_2_ oscillator exhibited novel oscillatory behaviours. As shown in Fig. 1c, the oscillator alters the phase‑space representation of chaotic temporal signals, suggesting an ability to extract information from complex inputs.

Finally, we inject speech signals into the oscillator at the EOC and utilize its capability to extract information in the frequency domain to perform speech classification tasks. The non-linear dynamics of a single device biased at the EOC were shown to be computationally equivalent to ∼10^4^ multiplication operations in neural networks. This work advances VO_2_ modelling theory with new experimental evidence, and bridges physical non-linearity with circuit dynamics. It enhances controllable computational complexity in artificial neurons via simple signal injection and accelerates the shift from traditional local passive devices to local active devices for dynamic physical computing.

## RESULTS

### Characterization of local active memristor

**Figure 2.**
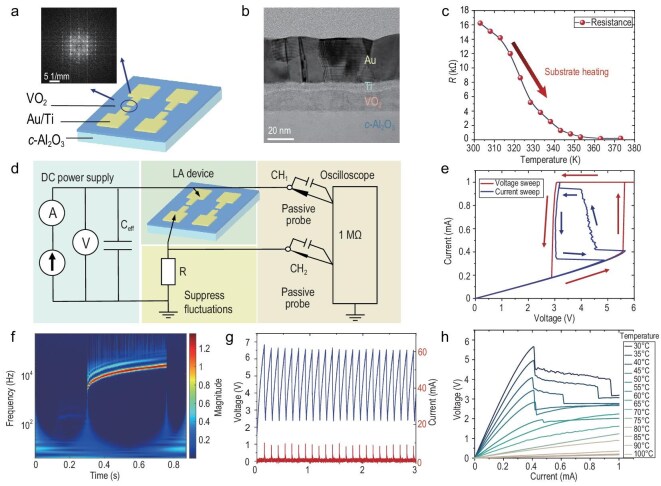
Electrical characterization of VO_2_ devices with locally active features. (a) Illustration of planar VO_2_ devices and the lattice diffraction pattern of VO_2_ thin film. (b) The TEM characterization image of the cross-section of the VO_2_ device. (c) The non-linear relationship between the VO_2_ device’s resistance and substrate temperature. (d) The experimental test circuit diagram. (e) The VO_2_ device’s response under static voltage sweep and static current sweep. (f) The VO_2_ device’s voltage response under current sweep in the frequency domain. (g) The oscillating behaviour and current spike firing behaviour when the VO_2_ device is biased at the NDR region. (h) The VO_2_ device’s I–V response at different substrate temperatures under quasi-static current sweep.

To construct a local active memristor, we employed the Mott material VO_2_, which exhibits insulator-to-metal transition dynamics near 340 K. This transition involves a temperature-sensitive electronic transition and a structural transformation from a low-temperature, low-symmetry monoclinic phase to a high-temperature, high-symmetry rutile phase [40]. VO_2_ thin films were epitaxially grown on c-Al_2_O_3_ substrates using pulsed laser deposition. The VO_2_ memristive devices feature a planar structure with the switching layer positioned between Au electrodes, as shown in Fig. 2a. Transmission electron microscopy (TEM) revealed the cross-sectional structure of VO_2_ thin films on substrates, as shown in Fig. 2b. Figure S1 also shows the microstructural and compositional characterization of the VO_2_ device, including cross-sectional scanning TEM (STEM) image and corresponding energy dispersive X-ray spectroscopy (EDS) mapping.

Figure 2c shows the device’s resistance–temperature characteristic. The experimental test circuit for the local active memristor is depicted in Fig. 2d. Under appropriate current bias, the memristor couples with external parasitic capacitances, giving rise to self‑sustained oscillations that are monitored by an oscilloscope. A series resistor is included to suppress fluctuations and stabilize the operating point. Figure S2 provides detailed circuit schematics and component values used in subsequent measurements. Under quasi-static bidirectional voltage sweep from 0 to 6 V, the device exhibits threshold switching. Under quasi-static current sweep from 0 to 1 mA, the device demonstrates a continuous negative differential resistance (NDR) region, as shown in Fig. 2e. The abrupt current rise during voltage sweep is attributed to thermal runaway, where current and device temperature increase synergistically, precluding an internal continuous transition. During current sweeping within the NDR region, the device oscillates, evidencing persistent dynamics indicative of local activity. The oscillatory behaviour and its Hopf bifurcation can be further resolved in the frequency domain via wavelet analysis, as shown in Fig. 2f. A distinct centre frequency is observed, which increases from 1 to 20 kHz as the bias current rises. The full time–domain response under static current sweep is provided in Fig. S3. In representative time windows, the device voltage oscillates while the device current exhibits spike‑like firings, as illustrated in Fig. 2g.

Furthermore, we varied the ambient temperature and performed quasi-static current sweeps, as shown in Fig. 2h. The NDR region is observed only below 318 K, which further substantiates that the NDR originates from the temperature-dependent phase transition dynamics. Notably, the onset of the NDR region remains nearly constant at approximately 0.4 mA across ambient temperatures, indicating that the initiation of NDR is driven by non‑thermal electronic processes. In particular, a current‑induced weakening of electron correlations delocalizes carriers in the insulating phase, thereby triggering the insulator–metal transition (IMT) [41,42]. Moreover, given the ease of integrating locally active memristors into measurement circuits, we also employed active probes to characterize both quasi-static I–V sweeps and oscillatory behaviours, as detailed in Figs S4–S5 and Note S2.

### Thermodynamics analysis of the local active memristor

To better bridge physical processes with emerging circuit theories and to account for the phenomena observed experimentally, a theoretical physical compact model of VO_2_ is necessary. The theoretical models of VO_2_ devices have been researched in recent years. Most of them are based on infinitesimal physical methods [43,44], which require substantial computing resources, or based on threshold voltage-driven regular oscillators [45,46], which oversimplify the behaviours of the local active oscillator. However, it is very complicated and hard to construct a VO_2_ compact active model due to the formation of high current density and high temperature channels in the uniform material. Also, the phase change process in VO_2_ devices simultaneously affects both conductance and thermal conductance functions. This is in contrast to NbO_2_, whose phase change temperature is much higher than the ambient temperature [20], meaning the NDR region can only be driven by the non-linearity of conductance. A recent compact model modelled the VO_2_ device as two parallel memristors and considered the channels’ non-linear transformation of conductivity and thermal conductivity during phase transition [47]. It is a huge leap, but it still complicates the model and ignores the possibility of an electrically induced phase transition. Besides, this method cannot keep the low resistance state of the device uniform during oscillation.

Here, we propose a compact and more simplified model based on Newton’s law of cooling and the first-order phase transition principle [48]. We simplified the conductivity and thermal conductivity into piecewise functions and introduced a constant obtained from experimental measurement to describe the dynamic switching. This model fits and interprets experimental data better and is suitable for SPICE circuit simulations.

Specifically, Chua’s theory of local activity underlies the entire capabilities and limitations of compact modelling of non-linear dynamic systems. Recent recontextualization of the local activity theory has streamlined its use as a design tool [22]. Compact models for memristors comprise physically reasonable transport models and relevant state-variable dynamic equations. The rich non-linear behaviours arise naturally from the interactions of these two ingredients. The electrothermal memristor model can be defined as:


(1) \begin{eqnarray*}
i = {G}_{el}\left( T \right)v,
\end{eqnarray*}

(2) \begin{eqnarray*}
\frac{{dT}}{{\ dt}} = \frac{1}{{{C}_{th}}}( {iv - {{G}_{th}}( T) \times( {T - {T}_0} ) + C} ),
\end{eqnarray*}

where *i, v* and *T* represent the device’s current, voltage and internal equivalent temperature, *T_0_* represents the ambient temperature, *G*_el_ denotes the device’s conductance, *G*_th_ represents the device’s thermal conductance and *C*_th_ is the device’s heat capacity. The device’s free energy density contains two contributions: the intrinsic bulk free energy density and the free energy density of excess free charge carriers. The complex phase-change dynamic in VO_2_ contains electron–lattice interaction and electron–electron interaction. The term *C* is introduced to effectively simplify dynamics and can refer to the latent heat effect of the first-order IMT in VO_2_ [48], which can fit the sudden change of the positive differential resistance (PDR) and NDR, and account for the abrupt change when the NDR region appears. By adding this constant, the improved model can fit the experiment well. We can only have one constant *C*_th_, one function of *G*_el_ and *G*_th_, and reduce the number of piecewise functions.

**Figure 3.**
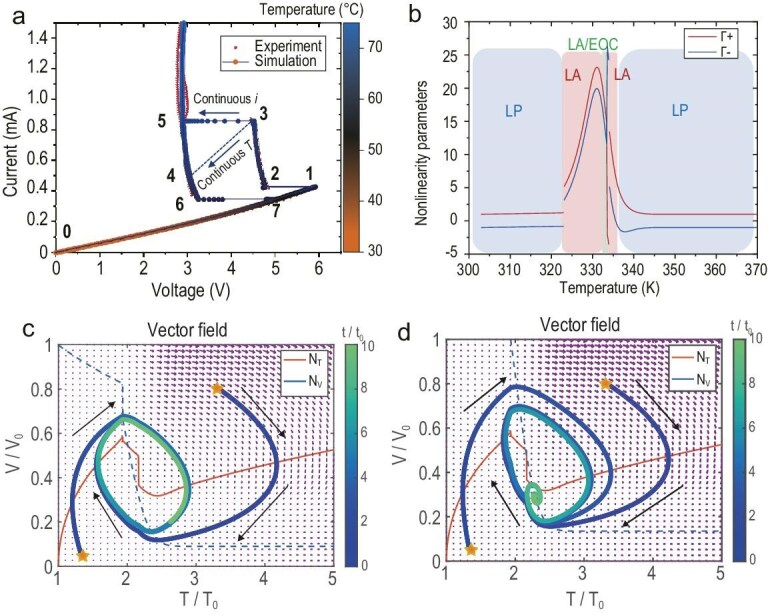
Analysis of theoretical model’s effect based on local active principle. (a) The experimental device’s I–V curve under static current sweep and theoretical model’s prediction results. (b) The device’s working region depends on its inner temperature based on local active theory, which can be divided into LP, LA and LA/EOC. (c) The device shows persistent oscillation behaviour in the phase plane. (d) The device shows decay behaviour in phase plane.

Based on the physical dynamic process, the device dynamics can be divided into eight special categories, as shown in Fig. 3a. These processes are detailed in Note S3. The key to the compact model based on local activity is the construction of the functions of *G*_el_(*T*) and *G_th_*(*T*). Only by considering both the non-linear transformations of conductance and thermal conductance can we accurately reproduce the NDR region that aligns with experimental results. We adopted the erf error function to fit *G*_th_ and *G*_el_ separately:


(3) \begin{eqnarray*}
R = \textit{erfc}\left( {\frac{{\left( {T - {T}_1} \right)}}{a}} \right) \times \left( {1 - \frac{T}{{{T}_c}}} \right) \times {R}_H + {R}_L,
\end{eqnarray*}

(4) \begin{eqnarray*}
\ {G}_{th} = {\mathrm{erf}}\left( {\frac{{T - {T}_2}}{b}} \right) \times {{G}_{th0}} + {G}_{th1},
\end{eqnarray*}

where *T*_1_, *T_2_, T_c_, a* and *b* are constants based on phase-change material characteristics, *R*_H_ is the high resistance state, and *R*_L_ is the low resistance state. Figure S6 shows the non-linearity curve of electronic conductance and thermal conductance with the device’s inner variable temperature changes. Figure S7 shows the detailed compact model simulation process, which is the most accurate model currently available. The parameters of *G*_el_ and *G*_th_ are mainly from fitting, and the fitting process is described in the Supplementary data (Methods and Table S2).

According to the theoretical model, we can divide the working region of VO_2_ devices into the local passive (LP) region, the local active (LA) region and the LA/EOC region [22].


(5) \begin{eqnarray*}
{\Gamma }_ + = \ \left( {{G}_{el}^{\,\,\,\prime}\ /\ {G}_{el} + \ {G}_{th}^{\,\,\,\prime}\ /\ {G}_{th}} \right) \times \left( {T\ - \ {T}_0} \right)\ + \ 1,
\end{eqnarray*}

(6) \begin{eqnarray*}
{\Gamma }_ - = \ \left( {{G}_{el}^{\,\,\,\prime} /\ {G}_{el} - \ {G}_{th}^{\,\,\,\prime}\ /\ {G}_{th}} \right)\times \left( {T\ - \ {T}_0} \right)\ -\ 1,
\end{eqnarray*}

(7) \begin{eqnarray*}
\textit{local}\ \textit{active}\!:\quad {\Gamma }_ + > \ 0\ \& \ {\Gamma }_ - > \ 0,
\end{eqnarray*}

(8) \begin{eqnarray*}
\textit{local}\ \textit{passive}\!:\quad {\Gamma }_ + > \ 0\ \& \ {\Gamma }_ - < \ 0.
\end{eqnarray*}

The working region of a single VO_2_ device is shown in Fig. 3b. The LA region begins from 323 K, which means the beginning of the EOC. Furthermore, phase plane and vector field analysis are powerful tools to analyse the behaviour of dynamic units. We define the zero line of temperature and voltage of N_T_: *dT/dt = 0* and N_v_: *dV/dt = 0*. The VO_2_ device’s behaviours depend on the intersection point of the temperature zero line and voltage zero line, as well as the external coupling circuit elements. Fixing a parallel capacitor, when the intersection point is in a local active region, the device shows persistent oscillation behaviour. All the initial states will converge to the limit cycle, as shown in Fig. 3c. When the intersection point is approaching the LP region, the device shows decay behaviour. All the initial states will converge to a working point, as shown in Fig. 3d. The vector field shows the direction in which the device will move when it is at an arbitrary point on the phase plane. Figure S8 shows the device’s voltage oscillation and current firing behaviour under a constant current bias, which is consistent with the observed phenomena in the experiment. The more dynamic cases upon different bias currents are shown in Fig. S9. These results explain that devices will have different dynamics (first LP, then LA and finally LP) in different bias currents. All the persistent dynamics are focused on the LA region.

### Dynamics of injection controlling at the critical EOC (CEOC)

In both experiments and theories, it has been proved that if the LA memristor is biased in the EOC region, it can induce oscillations by coupling with an appropriate external capacitance. Different oscillators can also be inter-coupled to perform complex computations for phase coding [49,50], solving combinatorial optimization problems [51,52]. Here, we will focus on exploring the complexity that a dynamic node can express. We further bias the device at the boundary between the PDR and NDR regions, which we call the CEOC point. At this point, we introduce an injection signal disturbance to excite more complex dynamics. Under this disturbance, the device alternates between the NDR and PDR regions, highlighting the importance of the coexistence of both regions in a passive component. If only the PDR region is present, the disturbance signal is simply superimposed on the bias. On the other hand, if only the NDR region is present, the signal disturbance is too little to affect the self-oscillation, and the device will continue self-oscillating with no obvious change.

**Figure 4.**
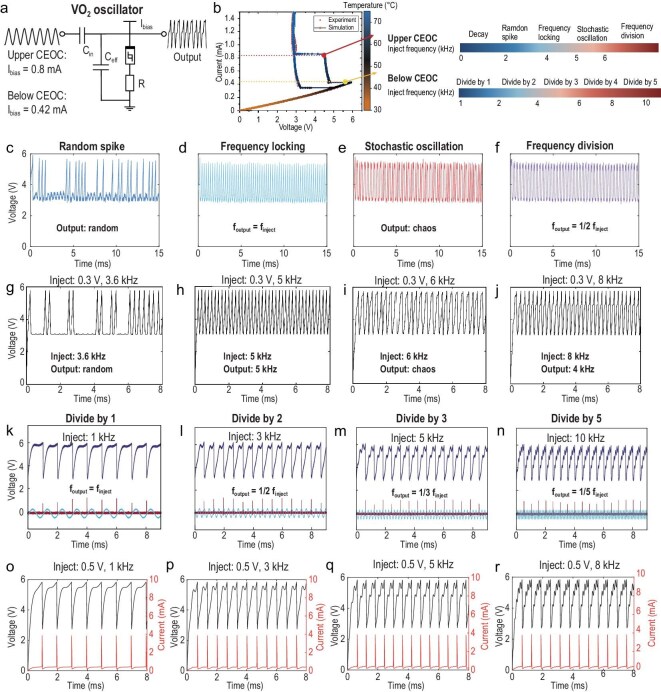
Dynamic behaviours of VO_2_ devices biased at CEOC under sinusoidal small signal excitation. (a) The circuit diagram and bias condition of the injection control behaviour. (b) Different biases will cause different responses to the injected signal. (c–f) The real electrical measurement results and (g–j) the model simulation results when biased at the upper CEOC (0.8 mA). (c and g) The random firing spike mode. (d and h) The frequency locking mode. (e and i) The stochastic oscillating mode due to thermal fluctuation. (f and j) The frequency modulation effect. (k–n) The real electrical measurement results and (o–r) the model simulation results when biased below CEOC (0.42 mA). The divided factors (f_inject_/f_output_) are (k and o) 1, (l and p) 2, (m and q) 3, (n and r) 5.

We set up an experiment, as shown in Fig. S10. The circuit diagram is shown in Fig. 4a. When injecting a voltage signal, the circuit function is as in Equation (9):


(9) \begin{eqnarray*}
&&\!\!\!\! \left( {{C}_{\textit e\!f\!f} + {C}_{in}} \right)\frac{{d{V}_{out}}}{{dt}} =\! \left( {{I}_{{bias}} - {I}_{{device}} + {C}_{in}\frac{{d{V}_{{inject}}}}{{dt}}} \right)\!,\\
&&\ \ \!\!\!\!
\end{eqnarray*}

where *C_eff_* is the effective parasitic capacitance in the VO_2_ oscillator’s circuit, *C*_in_ is the injection capacitance, *V*_inject_ is the injected voltage signal and *V*_out_ is the output voltage signal, *I*_bias_ is the current source to control the bias state and *I*_device_ is the current flowing through the VO_2_ device for effective electric thermal interaction. The current source keeps biasing the VO_2_ device on the CEOC point. while the voltage source applies a sinusoidal signal $V = \textit{Acos}( {2\pi ft} )$ with different frequencies through a series capacitance to the device. Equation (10) becomes as follows:


(10) \begin{eqnarray*}
&&\left( {{C}_{\textit e\!f\!f} + {C}_{in}} \right)\frac{{d{V}_{out}}}{{dt}}\\
&&\quad = \left( {{I}_{{bias}} - {I}_{{device}} + {C}_{in} \times 2\pi \textit{fAcos}( {2\pi ft})} \right).\\
\end{eqnarray*}

We then observe the voltage and current responses of the device. From the quasi-static I–V sweep, there are two CEOC points in the VO_2_ device. The current bias conditions and the corresponding behaviours are shown in Fig. 4b. As mentioned in Fig. 2h, the upper CEOC bias point will vary with the substrate temperature and the lower CEOC bias point will be constant within a certain temperature range.

If only considering the small parasitic capacitance, the device has an intrinsic frequency of oscillation. However, when considering the big parallel inject capacitance (15 nF), the device biased at CEOC will present decay dynamics and cannot oscillate. This indicates that an appropriate injection stimulation can make the oscillator work. We can adjust the amplitude of injection signals to make the device more easily swing between the LA region and LP region at the CEOC point in real electrical measurements. When the VO_2_ oscillator is biased at 0.8 mA (upper CEOC), as the frequency of the injection signal changes, the device’s voltage response undergoes continuous transformation, showing the non-linear response in the frequency domain, as shown in Fig. 4c–f. More detailed experimental results can be found in Figs S11–S14. The amplitude of the injected voltage signal is 0.3 V. When the injected signal frequency is lower than 2.5 kHz, the oscillator is not working and the VO_2_ device stays in high conductance state. When the injected signal frequency is 2.5–4 kHz, the oscillator exhibits random spiking with a gradually increasing probability, as shown in Fig. 4c. The relationship between the probability and the injected frequency is shown in Fig. S12, which can be used for true random number generation and probabilistic computing. The oscillator shows the frequency locking with the injection signal frequency from 4 to 5 kHz, as shown in Fig. 4d. The reason is that the integrated intensity of the injection signal is just enough to trigger oscillation in one cycle. It can be seen as a special intrinsic oscillation frequency at the CEOC. Furthermore, the injected signal affects the waveform of the oscillation and shows stochastic oscillation, as shown in Fig. 4e. As a hypothesis, we assume the source of the stochastic is the thermal fluctuation of the transition temperature in the device, which can be used for constructing chaotic oscillations. The cycle-to-cycle variation is amplified to different behaviours when biased at the CEOC point. In the simulation process, we added a random perturbation function on the transition temperature as cycle-to-cycle variation, which is amplified at the CEOC point. Finally, the effect of the injection signal will accumulate and trigger the VO_2_ device’s firing. Therefore, when the frequency of the injection signal is greater than 6 kHz, the oscillator behaviour shows the frequency modulation effect, which represents the integer division of injection frequency. Specifically, after precise integer division to the injection signal’s frequency, it is used as the frequency of device oscillation, as shown in Fig. 4f. The simulation results based on the proposed compact model also verified all the above experimental behaviours with high accuracy. Figure 4g and Fig. S15 verified the random spike behaviours. Figure 4h–i and Fig. S16 verified the frequency locking and stochastic oscillating behaviours. Figure 4j and Fig. S17 verified the frequency modulation behaviours.

When we bias the device at the stable point of the lower CEOC on 0.42 mA, we can observe different behaviours. The amplitude of the injection voltage signal is 0.5 V. The waveform is modulated into a square-like wave when the input signal is 1 kHz, and the oscillation frequency matches the input frequency. When the injection signal frequency is increasing, the oscillator’s frequency goes through divide by 2, 3, 5 on 3 kHz, 5 kHz, 10 kHz, as shown in Fig. 4k–n. The simulation results of these divided frequency behaviours are shown in Fig. 4o–r and Fig. S18. Figure S19 provides real photographs of the experimental setup and oscilloscope panel displays to prove that the above phenomena are both experimentally and theoretically verified. Therefore, all these complex dynamics can be simulated by the proposed compact model and indicate the VO_2_ device biased at the CEOC is capable of processing information in the frequency domain. If we bias the device at the middle of the EOC, according to local active theory, the small injection disturbances will always trigger persistent regular oscillations and it cannot show these various dynamic behaviours. When at a precise cycle round, the charge on the capacitor has reached the threshold. The device’s thermal runaway occurred and transitioned to a low resistance state. Therefore, the other part of the small signal will no longer have any significant impact until the oscillator automatically returns to the high resistance state. Furthermore, to verify that the injected signal only affects this current source oscillator, Fig. S20 shows the injection locking behaviour in the voltage-source-based oscillators, whose oscillating frequency is forced to be equivalent to the injection frequency.

### EOC oscillator computing for speech recognition

Temporal signals in real-world applications primarily originate from vibrations, making their frequency domain characteristics crucial. Moreover, the signal on time t and time t + 1 are correlated. Neural networks classify information through a chain reaction: one neuron after another, with each input undergoing a series of non-linear transformations. In speech recognition tasks, the same number will consistently trigger a similar chain reaction, even when pronounced by different speakers. Different numbers generate different chain reactions, thus enabling pattern recognition.

**Figure 5.**
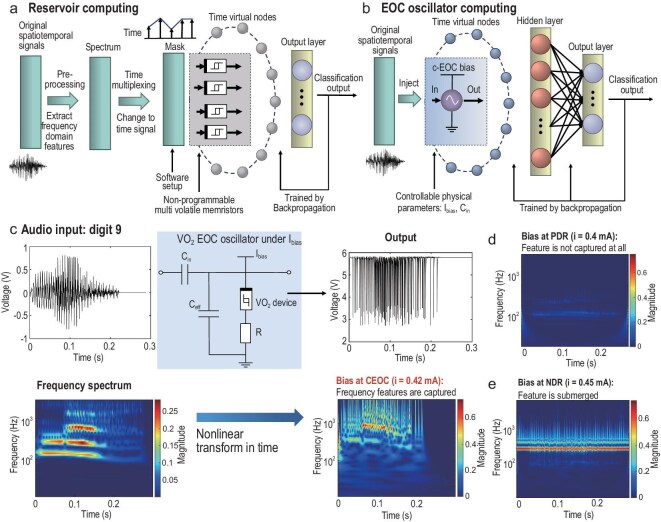
CEOC oscillator computing in speech recognition task. (a) The reservoir computing flow process diagram of the speech recognition task. (b) The EOC oscillator computing flow process diagram of the speech recognition task. The EOC oscillator can extract features from the frequency domain. (c) The detailed circuit implementation of EOC oscillator computing. (d) The frequency information disappeared due to the small signal attenuation effect when biased at PDR region. (e) The frequency information is submerged by the intrinsic oscillation when biased at NDR region.

From the above experimental demonstration, it is proved that VO_2_ EOC oscillators exhibit a frequency-specific continuous response to the injected sinusoidal signal with different frequencies. It implies that the EOC oscillators can directly extract features in the frequency domain and present differences in behavioural levels, showing computational superiority over other local passive memristors. For example, reservoir computing is now a popular information processing method for time series based on a volatile memristor’s decay dynamics. However, the reservoir cannot be adjusted. It can only process the temporal relationships of a series and has difficulty in handling the frequency characteristics due to the fixed time step. The reservoir computing is based on the monotonic non-linear decay dynamics; it almost has no frequency-sensitive capacity. So, for complex audio signal, the reservoir needs to preprocess in the frequency domain and then can process these pre-extracted features into a higher-dimensional space and then a classifier. For the spoken digit classification task, Fig. 5a shows the complete reservoir computing process. The original audio waveform needs to be first filtered into a spectrum with frequency channels per frame by using Lyon’s passive ear model [53]. The filtered signal then needs to go through a mask layer to reshape as a new time series, which contains frequency features. Also, it requires a significant amount of local passive devices due to the limited memory capacity of single devices.

To further verify its computational superiority for EOC oscillator computing, we also used a public spoken digit classification task [54] to compare with other existing computing methods. The dataset contains three people’s speech data to 10 digits in a sampling frequency of 8 kHz. The memristive oscillator plays a role as the low-power preprocessing unit for speech signal analysis.

For EOC oscillator computing, it has inner physical oscillation frequency, which means it is very sensitive to the nearby frequencies because they will cause different oscillation behaviours. The total computing process is shown in Fig. 5b. We set the injection capacitance as 20 nF to make the oscillator’s equivalent intrinsic frequency at the CEOC close to speech information. The normalized audio voltage signal can be directly injected into the CEOC VO_2_ oscillator biased at 0.42 mA. We can then observe and sample the continuous voltage signal output. Hereby, we sampled 1000 points on average as time virtual nodes. The time virtual node [55], originating in reservoir computing, is an effective method for reducing the large number of computing elements to a single non-linear node with delayed feedback. The excitations of a single physical node in response to the delayed signals can effectively act as a chain of virtual nodes [56]. It makes the node’s inner states and the input signal non-linearly coupled. Diverse responses can be obtained from the virtual nodes. According to generalized embedding theories, using time delays can reduce network sizes significantly, and will achieve dynamics reconstruction with a single dynamic device [57]. Finally, a multi-layer perceptron was applied to classify the output vector of the virtual nodes.

The detailed EOC oscillator circuit with input audio waveform and output voltage waveform is shown in Fig. 5c. The injected input will disturb the device, causing it to swing between different regions, accumulating and amplifying small input signals in the LA region, while attenuating noisy signals in the LP region. The injected input will trigger the chain reaction in the CEOC memristive oscillator’s time virtual nodes, whose future state depends on its past state. As a result, the oscillator can capture the frequency-concentrated components of speech information as features and express them as non-linear oscillations in the time domain. Otherwise, the LP region can only filter signals in simple frequency bands and the LA region can only amplify signal fluctuations and convert them into regular oscillations, drowning out the injected information. In conclusion, the oscillator biased at the CEOC state can strengthen the chain reaction by swinging between LA and LP, performing a non-linear time transformation to make the signal more accessible to simple perception.

Through wavelet transform, we also display their corresponding frequency domain diagrams. It shows that the high-frequency and high-amplitude parts of the original signal correspond to the high-frequency part of the output signal. The CEOC oscillator can filter the useless small amplitude information and accumulate the stimuli over a period of time on the charge of a capacitor, and finally fire and refresh when the threshold voltage is reached. The current bias needs to be fine-tuned. If the VO_2_ oscillator is biased at the normal PDR region or NDR region, the information will disappear through dissipation (as shown in Fig. 5d) or be submerged by the self-oscillation of the oscillator (as shown in Fig. 5e). The detailed algorithm process is described in the Methods. The typical digital output spectrogram is shown in Figs S21–S23, which can help to intuitively understand the frequency domain features extracted from each number’s corresponding output waveforms. It can be seen that the spoken digits have some specific frequency characteristics that are clear in the frequency domain but not sensibly in the time domain. These features cannot be extracted by local passive devices because they can only capture the discrete sequence size values. Only local active devices that can respond differently to different frequencies can capture these features. The high-frequency and high-amplitude parts, the oscillator tend to fire more densely.

**Figure 6.**
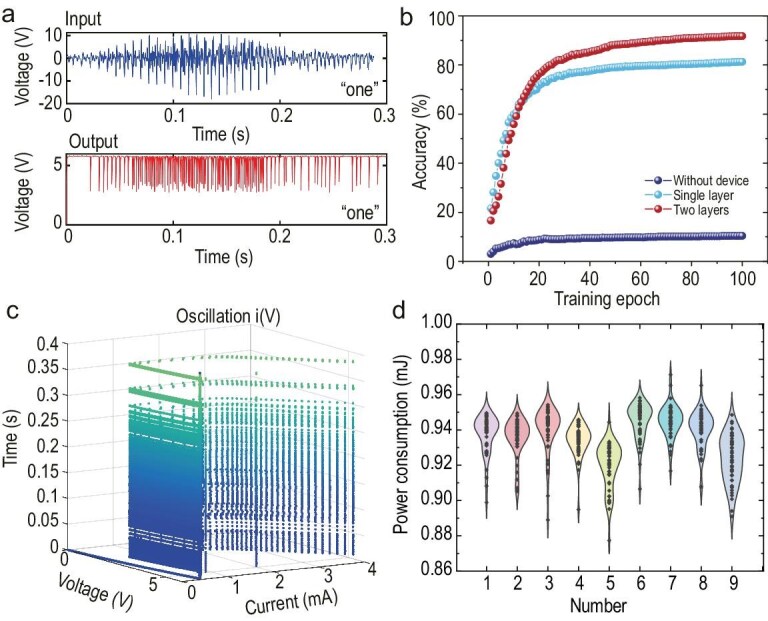
The performance of CEOC oscillator computing in speech recognition task. (a) The original audio signal of digit one and the output of the CEOC oscillator. (b) The classification results of three cases: without device, single layer for readout, two layers for readout. (c) The current and voltage evolution map of the CEOC oscillator while receiving speech signals. (d) The power consumption distribution of 10 speech numbers.

Figure 6a shows the comparison of the original audio signal of digit one and the output of the CEOC oscillator. As shown in Fig. 6b, the classification accuracy achieved by a single-layer perceptron is 80%, while a two-layer perceptron achieves 92%. As a control group, we directly collected 1000 points from raw speech data and input them into a multi-layer perceptron, whose accuracy is only 11%. The deep neural network, which is composed of two convolutional layers and two fully connected layers, achieves an accuracy of 94%. Therefore, the single CEOC VO_2_ oscillator can be equivalent to two convolutional layers in this case, reducing 10^5^ multiplication operations in conventional methods. Note S4 describes the detailed structure of the convolutional neural network (CNN). This application demonstrates the significant information processing capability of CEOC devices in the continuous time domain.

More importantly, the frequency information is represented by the self-oscillator’s trajectory in the phase space, as shown in Fig. 6c. This means that utilizing physical dynamics to process information will depend on trajectories and will not introduce significant additional power consumption compared to the normal self-oscillation. It is because the computing ability is totally from the special expression under physical differential equations. The energy computing method is described in Note S5. Figure 6d shows the power consumption for the CEOC oscillator to each digit speech input in 0.5 s. If without input, the power consumption for self-oscillation is 0.97 mJ. If with spoken information input, the power consumption is 0.88–0.97 mJ. It proves that the frequency domain feature extraction in a CEOC oscillator does not introduce additional power consumption. Table 1 and Notes S6–S7 compare the proposed CEOC oscillator computing with other existing computing methods [8,58,59]. Here we use a local active memristor working at the EOC. Just one oscillator’s virtual nodes can replace the massive local passive devices. The computing capacity includes frequency domain feature extraction and non-linear response, showcasing the powerful advantages of utilizing the physical dynamics of CEOC memristive devices for computation. This system truly processes data continuously from physical dynamics without finite time steps. The max processing speed is restricted by the oscillator’s intrinsic limit oscillation frequency due to thermal relaxation.

**Table 1.** Comparison with other physical computing methods.

	Deep learning accelerating	Reservoir computing	EOC oscillator computing (this work)
Computational source	Linear Ohm’s law	Non-linear decay dynamic	EOC oscillator dynamic
Devices for preprocessing	N*M	1*N	1
Preprocessing method	Spectrogram and cochlear models	Spectrogram and cochlear models	In local active device processing
Network architecture	Preprocessing 2-layer CNN 2-layer FC	Preprocessing reservoir 1-layer FC	1 device 2-layer FC
Power consumption	(CPU) 13.7 W [59] (FPGA) 83 mW [59]	15 mW [8] 3.2 mW [58]	1.76–1.94 mW
Speed/time step	(CPU) 4 μs/time step [59] (FPGA) 1.73 μs/time step [59]	10 μs/time step [8] 120 μs/time step [58]	Physical continuously

## DISCUSSION

Overall, in this work, we have experimentally summarized the local active characteristics of VO_2_ Mott devices and proposed a compact model based on the local active principle for dynamics analysis. Furthermore, we have demonstrated how complex oscillation behaviours can be constructed when a local active device biased at the CEOC point receives injection signals with different frequencies. On these grounds, we further explored the device’s ability to process temporal information based on CEOC dynamics and found it has powerful frequency domain feature extraction capability, surpassing the existing local passive memristors. This work reveals a brand new approach to physical dynamic computing based on local active memristors, applying critical physical features to information processing.

This dynamic EOC computing paradigm can be biased stable and has robustness to environmental temperature. The information processing ability is displayed in its dynamic trajectory and will not introduce additional large power consumption compared to its self-oscillation behaviour. A single local active memristive device can handle the speech recognition’s preprocessing, showing superiority in both computing capacity and power consumption. This work breaks through the paradigm shift from traditional local passive devices to local active devices, paving the way for bridging the physical non-linear characteristics, circuit dynamics and computational theory towards dynamic neuromorphic computing.

## METHODS

The methods used are described in the Supplementary data.

## Supplementary Material

nwaf546_Supplemental_File

## Data Availability

All data supporting this study and its findings are available in the article and the Supplementary data. The codes used for the simulations are available from the corresponding author upon reasonable request.
