# Supplementary material for: Local active memristive oscillator enables controllable complex behaviours and frequency domain extraction
Source: Natl Sci Rev. 2025 Dec 8;13(2):nwaf546. doi: 10.1093/nsr/nwaf546 (PMC12831030; doi:10.1093/nsr/nwaf546)
Supplement: nwaf546_Supplemental_File [file nwaf546_supplemental_file.pdf]

Supplementary information

Local active Memristive oscillator enables controllable complex behaviours and frequency domain extraction

Yanghao Wang[1], *Pek Jun Tiw*[1], *Yuheng Liu*[1], *Yaoyu Tao*[2], *Teng Zhang*[1,5*] *and Yuchao Yang*[1,2,3,4*]

[1] Beijing Advanced Innovation Center for Integrated Circuits, School of Integrated Circuits, Peking University, Beijing 100871, China.

[2] Institute of Artificial Intelligence, Peking University, Beijing 100871, China.

[3] Guangdong Provincial Key Laboratory of In-Memory Computing Chips, School of Electronic and Computer Engineering, Peking University, Shenzhen 518055, China.

[4] Center for Brain Inspired Intelligence, Chinese Institute for Brain Research (CIBR), Beijing, 102206, China.

[5] State Key Laboratory of Multimedia Information Processing, School of Computer Science, Peking University, Beijing, 100871, China.

Methods

Device Fabrication.

The 20 nm VO_2 films were grown on c-Al_2O_3 substrates in an epitaxial manner by pulsed-laser deposition (PLD) technique using a 308-nm XeCl excimer laser operated at an energy density of about 1 J/cm^2 and a repetition rate of 3 Hz. The VO_2 films were deposited at 530 ℃ in a flowing oxygen atmosphere at the oxygen pressure of 2.0 Pa. Then, the films were cooled down to room temperature at the speed of 20 ℃/min. The deposition rate of VO_2 thin films was calibrated by X-ray Reflection (XRR). The electrodes, which are composed of Au (40 nm) and Ti (5 nm) with a distance of 400 nm, were patterned with electron beam lithography (EBL) along with electron beam evaporation and lift-off. More than two hundred devices were fabricated.

Electrical Measurement.

The electrical measurements, including the DC test of the VO_2 memristor and the pulse test of the oscillators, are conducted using an Agilent B1500A semiconductor parameter analyzer. The Agilent B1500A is also employed to provide the long current pulse as the bias during the experiments of injects. The inject voltage signal is provided from RIGOL DG4202 function waveform generator. All the oscillatory waveforms are captured by the KEYSIGHT DSOS404A digital storage oscilloscope. We mainly use passive probes for measurement. The detailed circuit diagram is shown in Supplementary Fig. 2. As a comparison, we also use active probes

(amplifier Keysight MX0025A and probe head MX0100A for measurement, as shown in Supplementary Fig. 3-4. The real testing circuit picture is shown in Supplementary Fig. 19.

Model Simulation

The model simulations are based on MATLAB R2022b. We construct the entire model by segmenting and fitting the data obtained from electrical testing. And the further local active analysis and small signal inject prediction are performed corresponding to the experimental test results. The detailed simulation process and parameters are explained in Supplementary Note.1. The VO_2 model is complicated in the nonlinear function of G_{el}-T and G_{th}-T. We first get some G_{el}-T parameters from the R-T static sweep, then we adjust the G_{th} function's parameters to fit the IV quasi-static sweep. Since the G_{th} is hard to measure, we assume the G_{th} is a two-stage nonlinear function. Stage 1: When device's temperature is less than 58 ℃, the phase change doesn't occur and the G_{th} is assumed as a constant G_{th1}. When temperature is higher than 58 ℃, we adjust parameters G_{th0} and b to fit the IV quasi-static sweep. Stage 2: When temperature is higher than 65 ℃, which we assume is the box-hysteresis temperature, then we keep G_{el} as the same and adjust the G_{th} parameters: $G_{th1'}$, $G_{th0'}$.

Audio Recognition

We use the Free Spoken Digit Dataset (FSDD) dataset v1.0.8. It is an audio dataset consisting of recordings of spoken digits in wav files at 8 kHz. For each digit, different people repeat 50 times and the duration is distributed between 0.4-1 second. When injecting into the CEOC VO_2 oscillator, the signal is first normalized to proper range (0-1V) and then we observe the continuous voltage signal output. The inject capacitance is 20 nF and the parallel capacitance is 5 nF. We sample 1000 points in average as time virtual node. Then the new data can be classified by multi-layer perceptron, which is trained by backpropagation. We use 1200 pieces of data as training dataset and 300 pieces of data as validation dataset. The accuracy is 80% in single layer perceptron (1000 neurons in input layer, 10 neurons in output layer) and 92% in two layer perceptron (1000 neurons in input layer, 100 neurons in hidden layer, 10 neurons in output layer). As a control, we compared directly collecting 1000 points from raw speech data and inputting them into a multi-layer perceptron, whose accuracy is only 11%. The CNN result is based on open-source codes on Kaggle for this Free-Spoken Digit Dataset. The detailed parameters of the CNN are shown in Supplementary Note 2. Besides, the reservoir computing rebased on memristor's nonlinear decay dynamic is also from the existing work on the same speech recognition task. This fairly demonstrates the information processing capability of chaotic edge devices in the continuous time domain. The power consumption evaluation is presented in Supplementary Note.3-4.

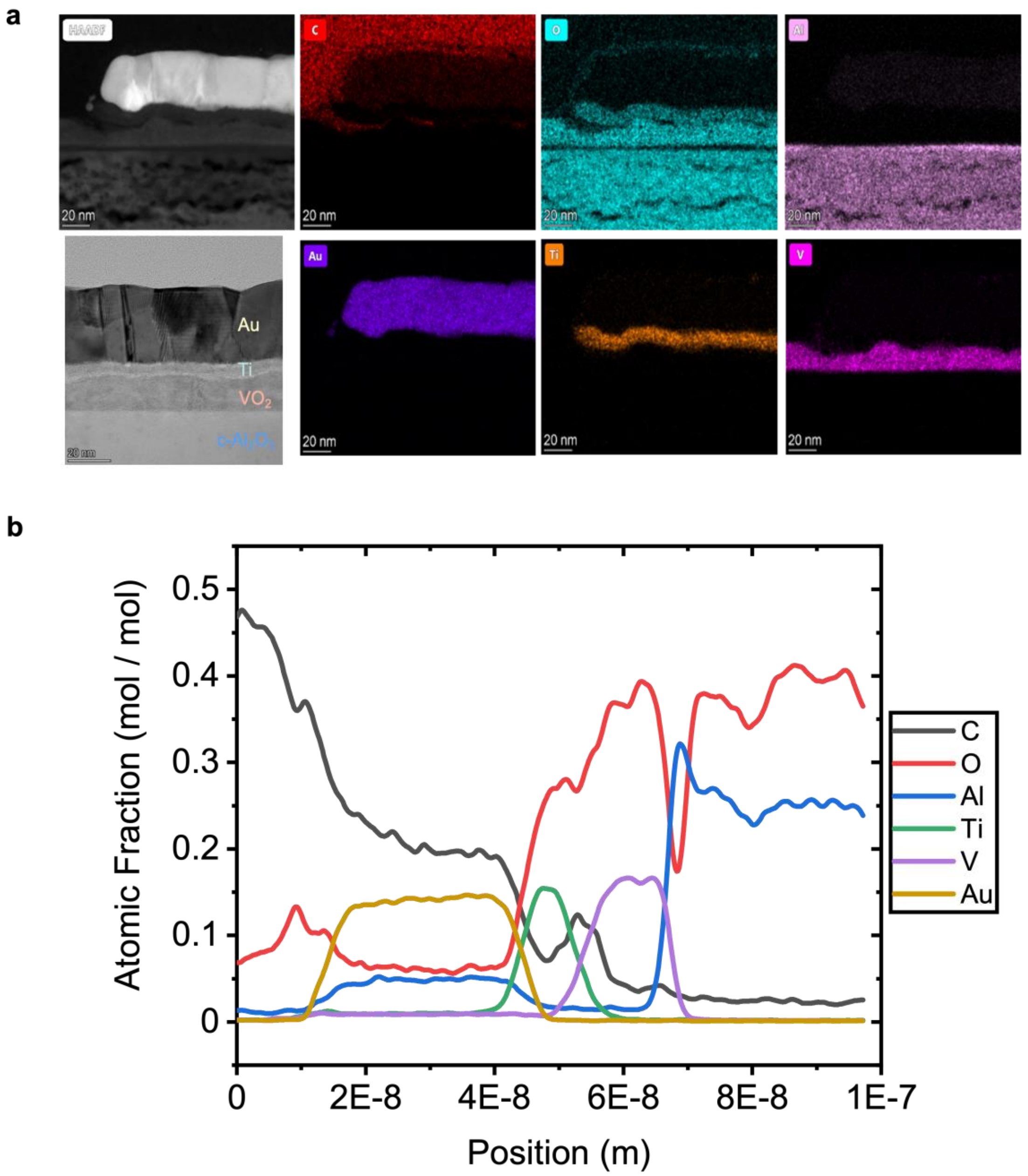

Supplementary Figure 1. Microstructural and compositional characterization of VO_2 device. (a) Cross-sectional STEM image and corresponding EDS mapping of O, V, Au, Ti elements in the device. (b) The relationship between atomic fraction and position in EDS line scanning.

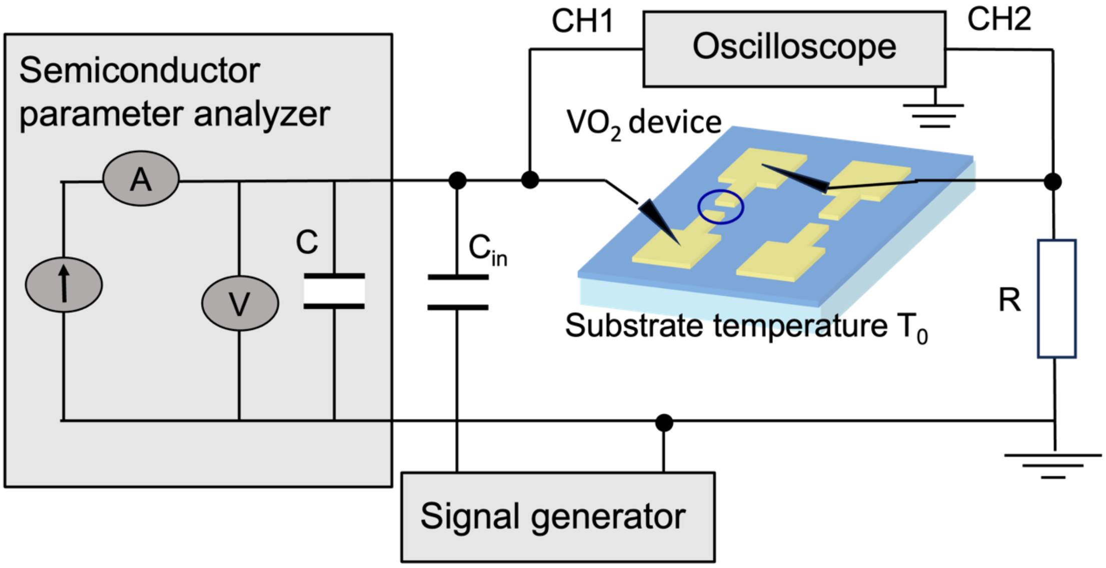

Supplementary Figure 2. Test circuit diagram of injection control. The test circuit includes a semiconductor parameter analyzer as a current source, a signal generator as the injected signal source, an oscilloscope for voltage measurement and a series resistor (100 ohm) for current measurement and to suppress fluctuations during quasi-static scanning. The internal resistance of the oscilloscope is 1 Mohm and passive probes are used here. The channel 1 (CH1) of the oscilloscope is for memristor and channel 2 (CH2) is for the series resistor.

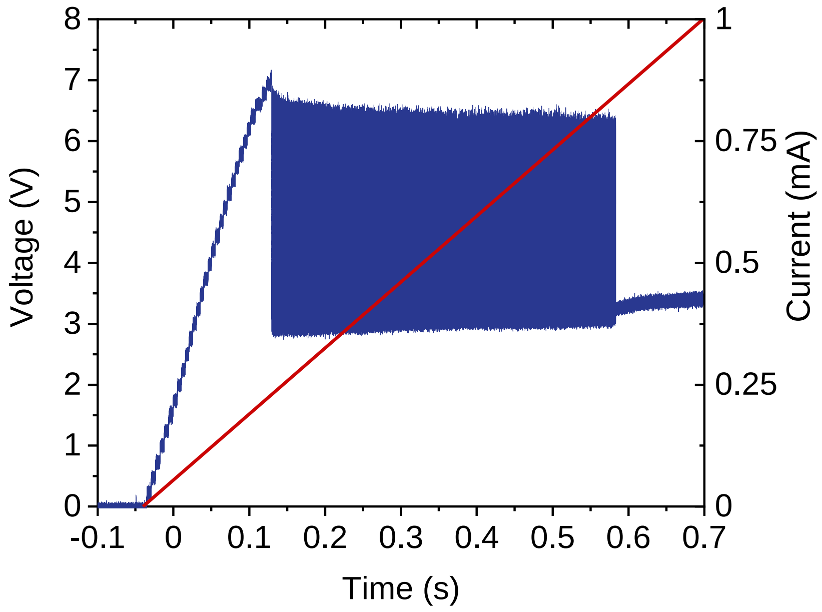

Supplementary Figure 3. The device response under static current sweep in time domain. The red curve is the driven current from 0-1 mA and the blue curve is the device response. It will oscillate from t = 0.12 s to t = 0.58 s.

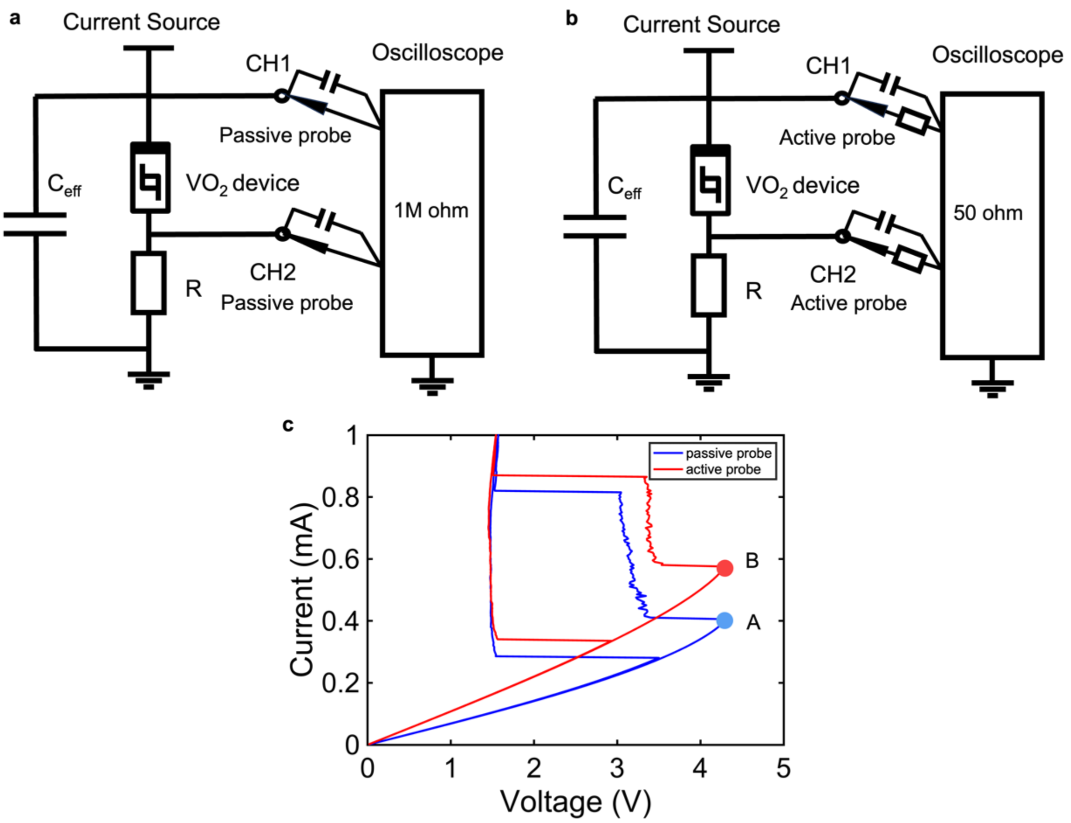

Supplementary Figure 4. The difference between the passive probe and the active probe of the oscilloscope. (a) The test circuit of using the passive probe (relatively high parasitic capacitance, low impedance) when the oscilloscope has 1M ohm internal resistance. (b) The test circuit of using the active probe (relatively low parasitic capacitance, high impedance) when the oscilloscope has 50 ohm internal resistance. (c) The quasi-static IV sweep results of the same devices using two probes. The active probe consists of amplifier Keysight MX0025A and probe head MX0100A. It has 25 kohm / 0.17 pF single-ended input impedance. Therefore, this type of active probe will introduce additional parallel resistors (25 kohm) in our experiments. The resistance of point A is 10710 ohm and point B is 7460 ohm. 10710 ohm // 25 kohm = 7498 ohm.

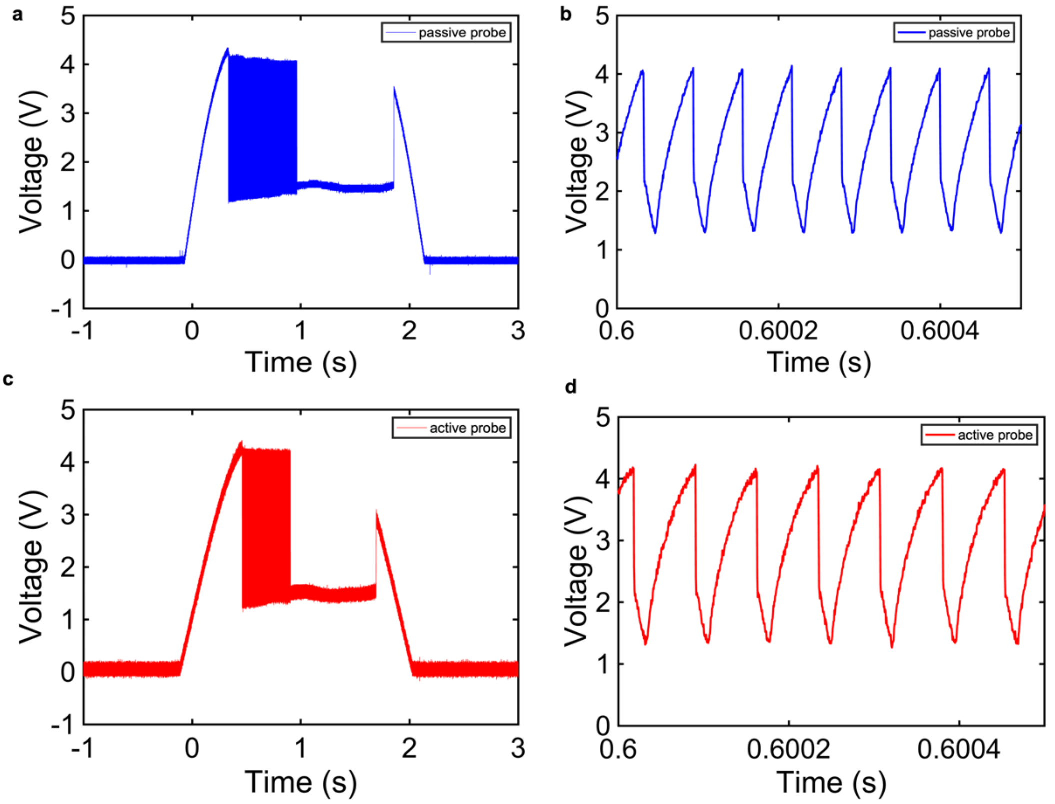

Supplementary Figure 5. The oscillation behaviour during iv sweep through passive probe and active probe. (a) The VO_2 device's voltage response under current sweep in the time domain through passive probe. (b) The VO_2 device's voltage oscillation under current = 0.6 mA through passive probe. (c) The VO_2 device's voltage response under current sweep in the time domain through active probe. (d) The VO_2 device's voltage oscillation under current = 0.6 mA through active probe. The difference is mainly from the 25k ohm parallel input impedance of active probe.

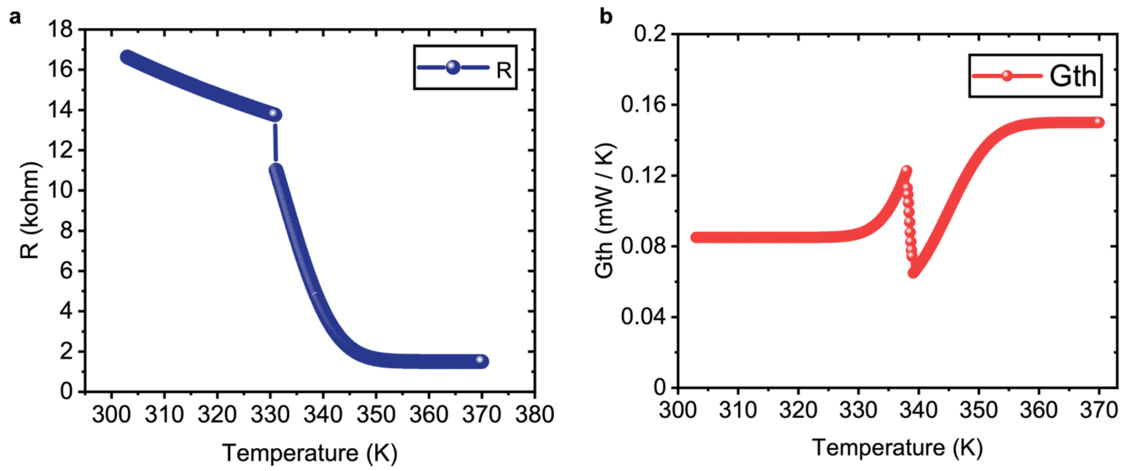

Supplementary Figure 6. Equivalent conductivity and thermal conductivity vary with device’s state variables from parameters fitting based on the model. (a) the resistance decreases as the device's inner temperature rises. The sudden change is because of the alterations in the conductive channel area (b) the thermal conductivity first increases, then suddenly decreases and finally rises to a higher state. The sudden change is caused by the device’s phase transition. The detailed fitting process is expressed in Methods.

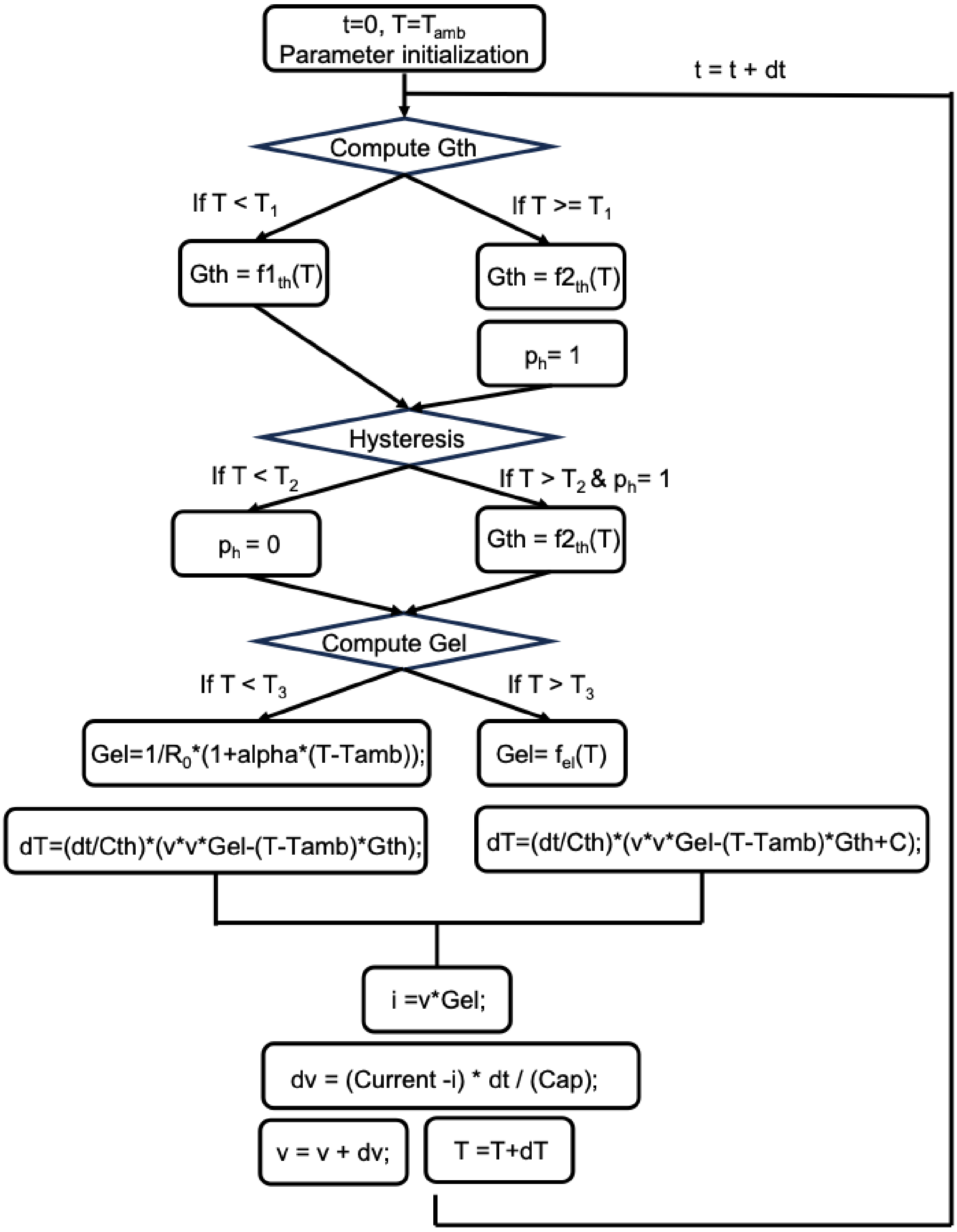

Supplementary Figure 7. The computing process of the proposed VO_2 compact model. The corresponding code is open-sourced as MATLAB codes. The detailed parameters are shown in Supplementary Table 2.

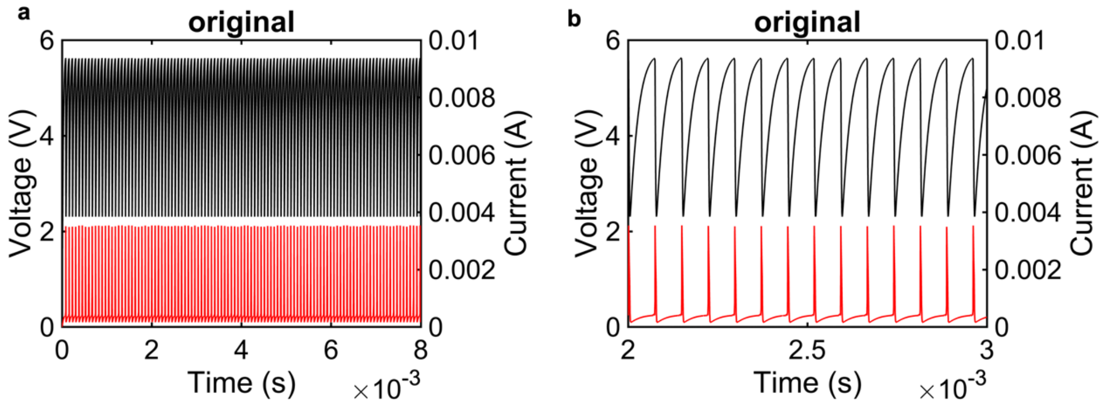

Supplementary Figure 8. The simulation of voltage oscillation and current spiking under current = 0.42 mA (around CEOC point). (a) The voltage oscillation and current spiking behaviour in 8 ms time scale. (b) The voltage oscillation and current spiking behaviour in 1 ms time scale. The oscillation frequency is 13.6 kHz.

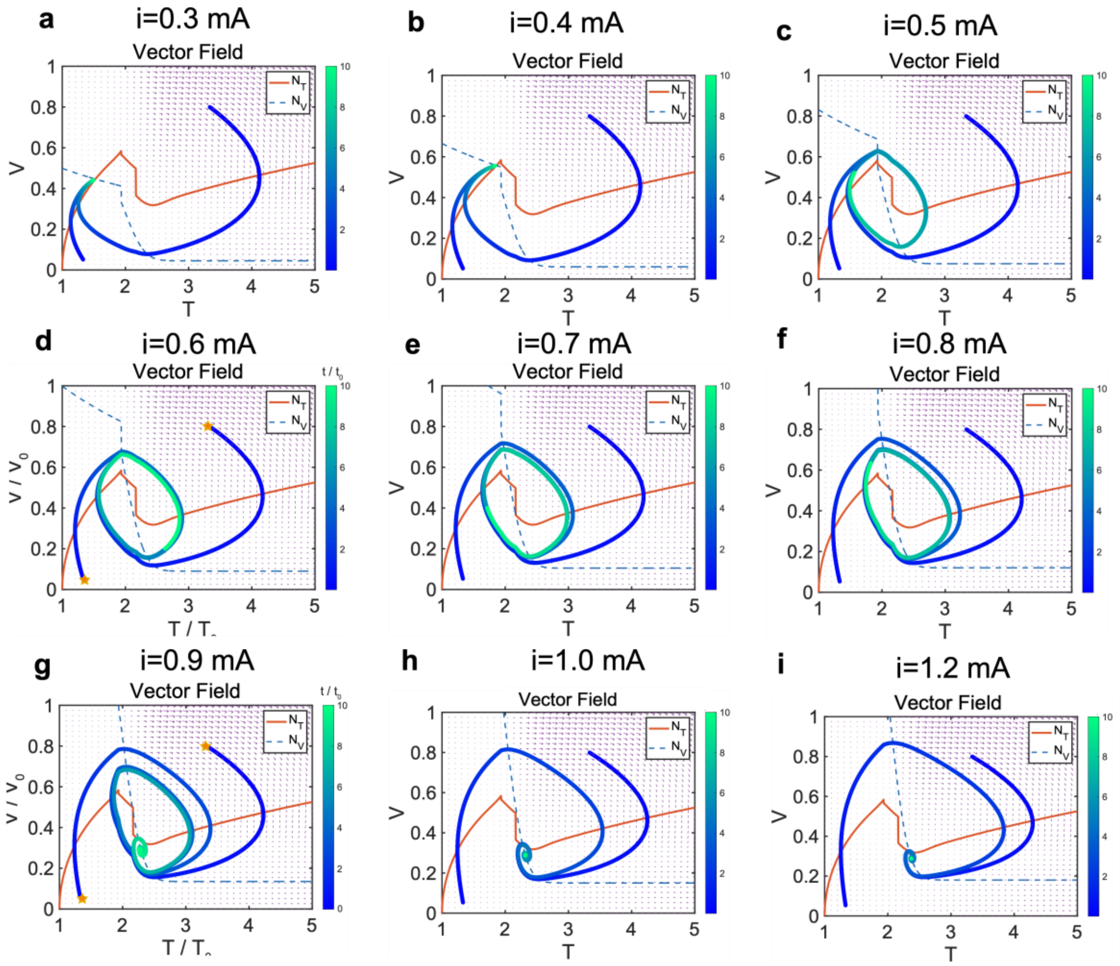

Supplementary Figure 9. The phase plane behaviour when device is biased at different currents. Local passive state, decay dynamic: (a) i=0.3 mA and (b) i=0.4 mA. Local active state, limit cycle as oscillating: (c) i=0.5 mA, (d) i=0.6 mA, (e) i=0.7 mA, and (f) i=0.8 mA. Local passive state, decay dynamic: (g) i=0.9 mA, (h) i=1.0 mA, and (i) i=1.2 mA.

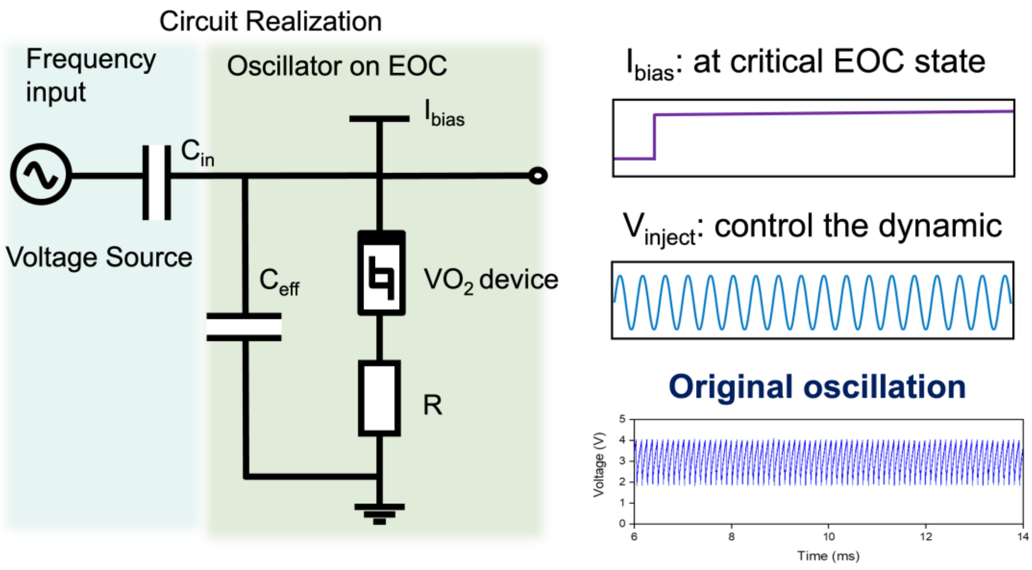

Supplementary Figure 10. Circuit diagram and signal setup for injection control. The source I_{bias} is a constant current to bias the device at the critical EOC state. The source V_{inject} is a sine signal with different frequencies and the typical peak-to-peak voltage amplitude can be 0.1V-1V. The VO_2 oscillator will self-oscillate without injected signal when biased at EOC.

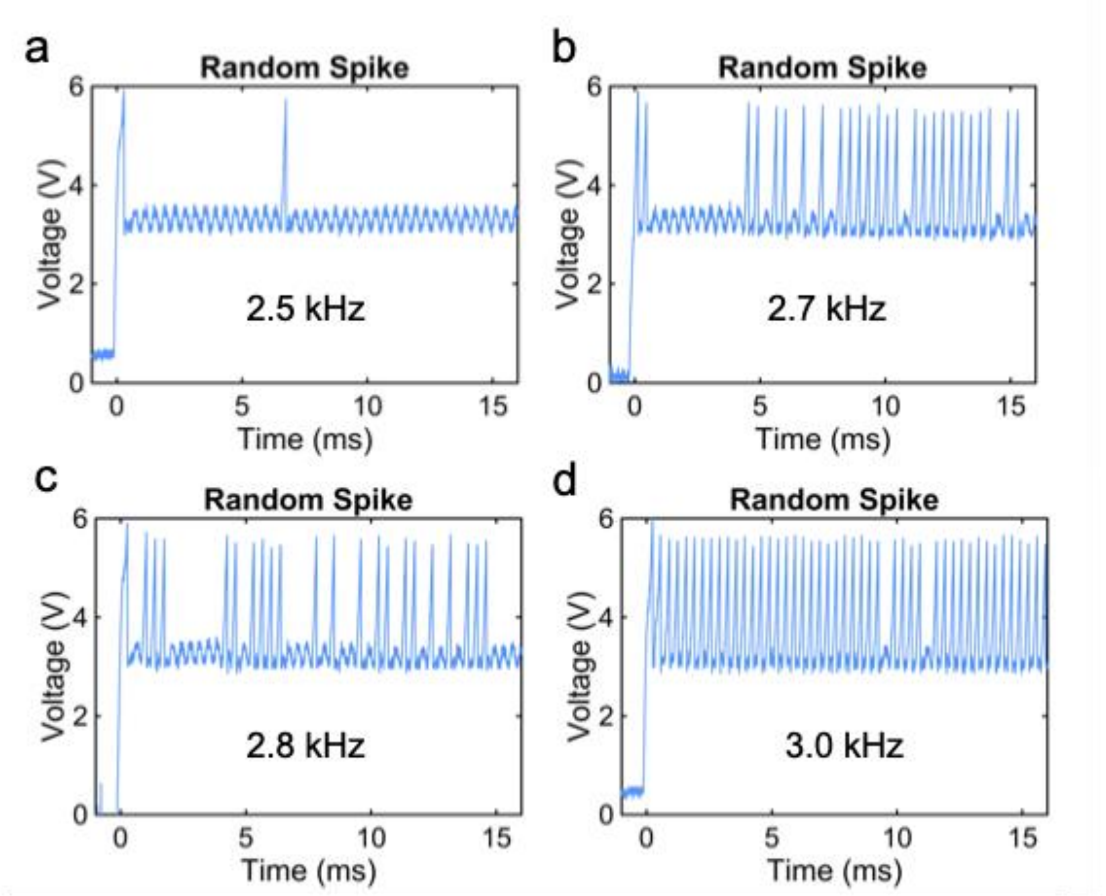

Supplementary Figure 11. The real electrical measurement results when the injection frequency is (a) 2.5kHz (b) 2.7 kHz (c) 2.8 kHz (d) 3.0 kHz under 0.8 mA bias.

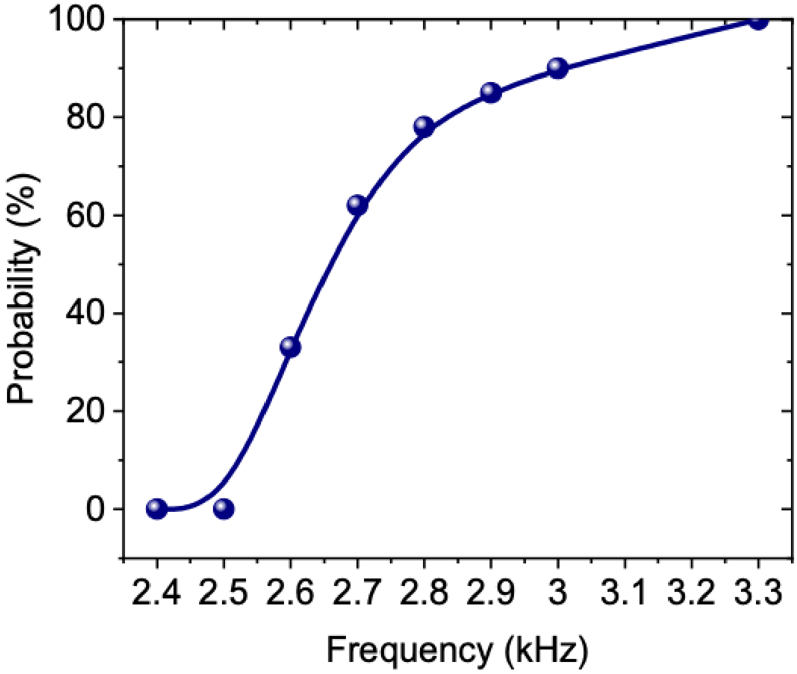

Supplementary Figure 12. The probability of random oscillations increases with the frequency of injected signals.

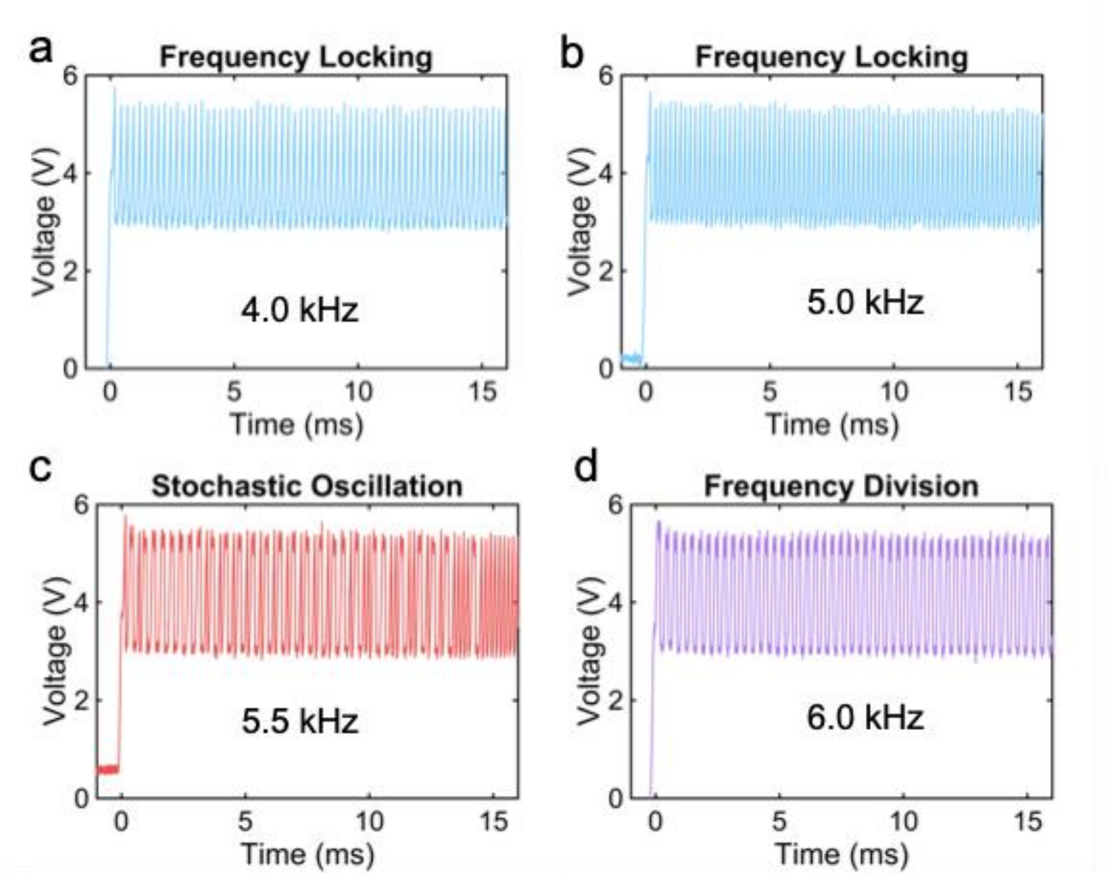

Supplementary Figure 13. The real electrical measurement results when the injection frequency is (a) 4.0kHz (b) 5.0 kHz (c) 5.5 kHz (d) 6.0 kHz under 0.8 mA bias.

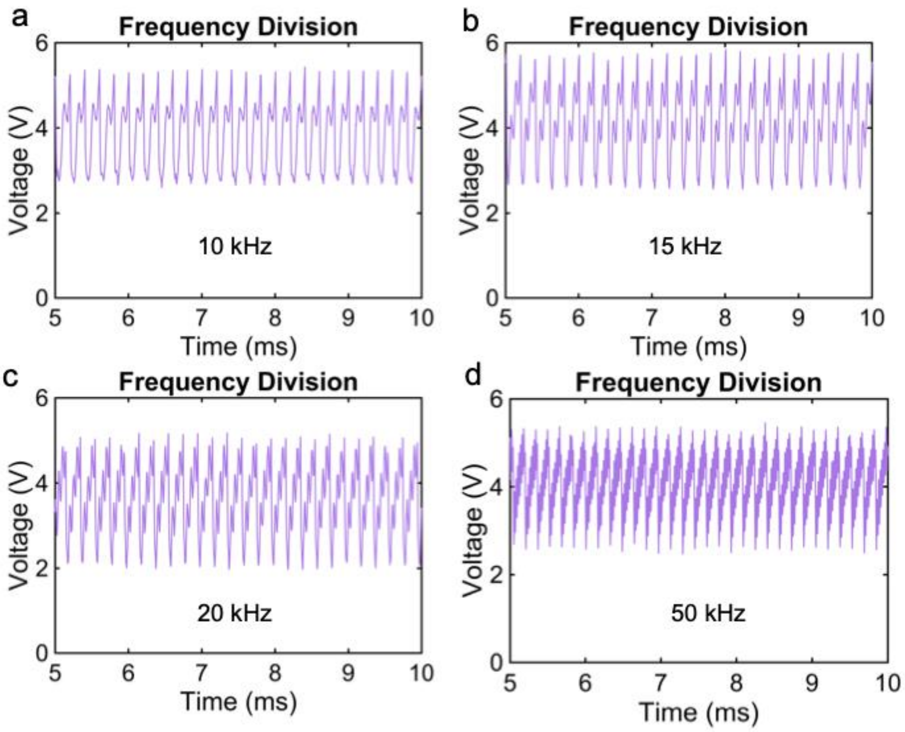

Supplementary Figure 14. The real electrical measurement results when the injection frequency is (a) 10 kHz (b) 15 kHz (c) 20 kHz (d) 50 kHz under 0.8 mA bias.

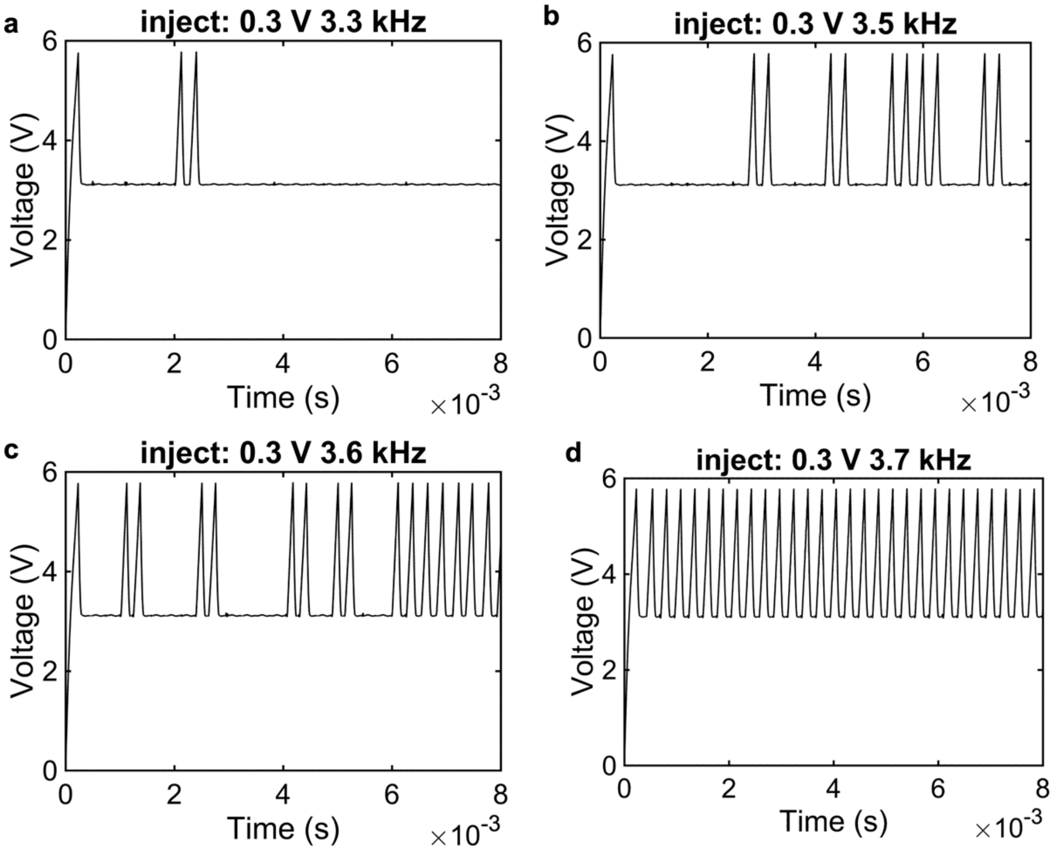

Supplementary Figure 15. The simulation results of random spiking behaviour. The bias current is 0.8 mA. The inject amplitude is 0.3 V. The inject capacitance is 20 nF. The inject frequency is (a) 3.3 kHz, (b) 3.5 kHz, (c) 3.6 kHz, (d) 3.7 kHz.

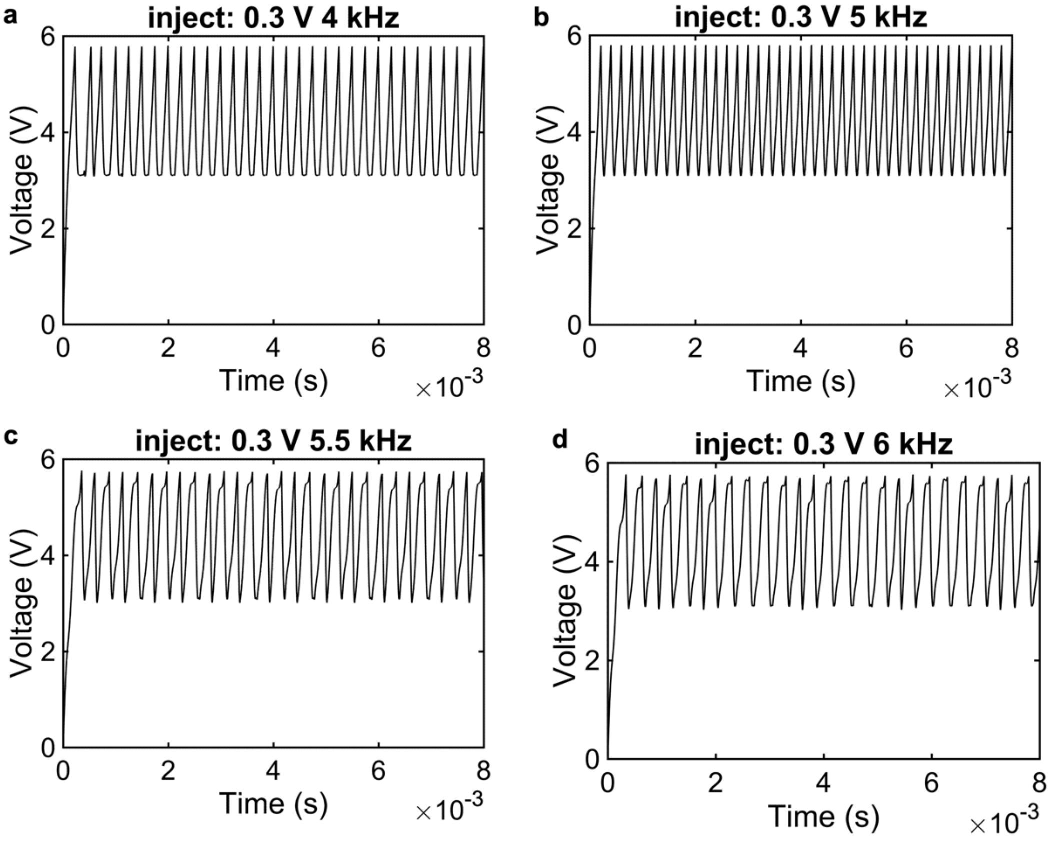

Supplementary Figure 16. The simulation results of frequency locking behaviour (a)&(b) and stochastic oscillation behaviour (c)&(d). The bias current is 0.8 mA. The inject amplitude is 0.3 V. The inject capacitance is 20 nF. The inject frequency is (a) 4 kHz, (b) 5 kHz, (c) 5.5 kHz, (d) 6 kHz.

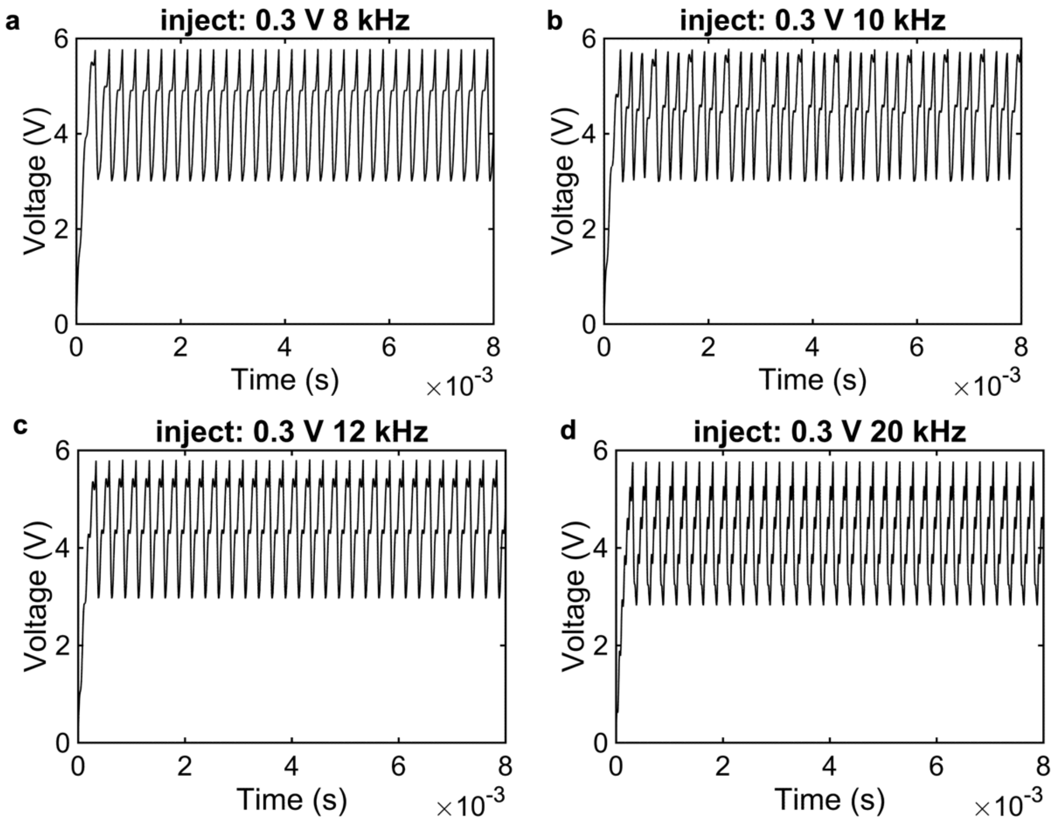

Supplementary Figure 17. The simulation results of frequency modulation behaviour (a)&(c)&(d) and the middle transitional condition (b). The bias current is 0.8 mA. The inject amplitude is 0.3 V. The inject capacitance is 20 nF. The inject frequency is (a) 8 kHz, (b) 10 kHz, (c) 12 kHz, (d) 20 kHz.

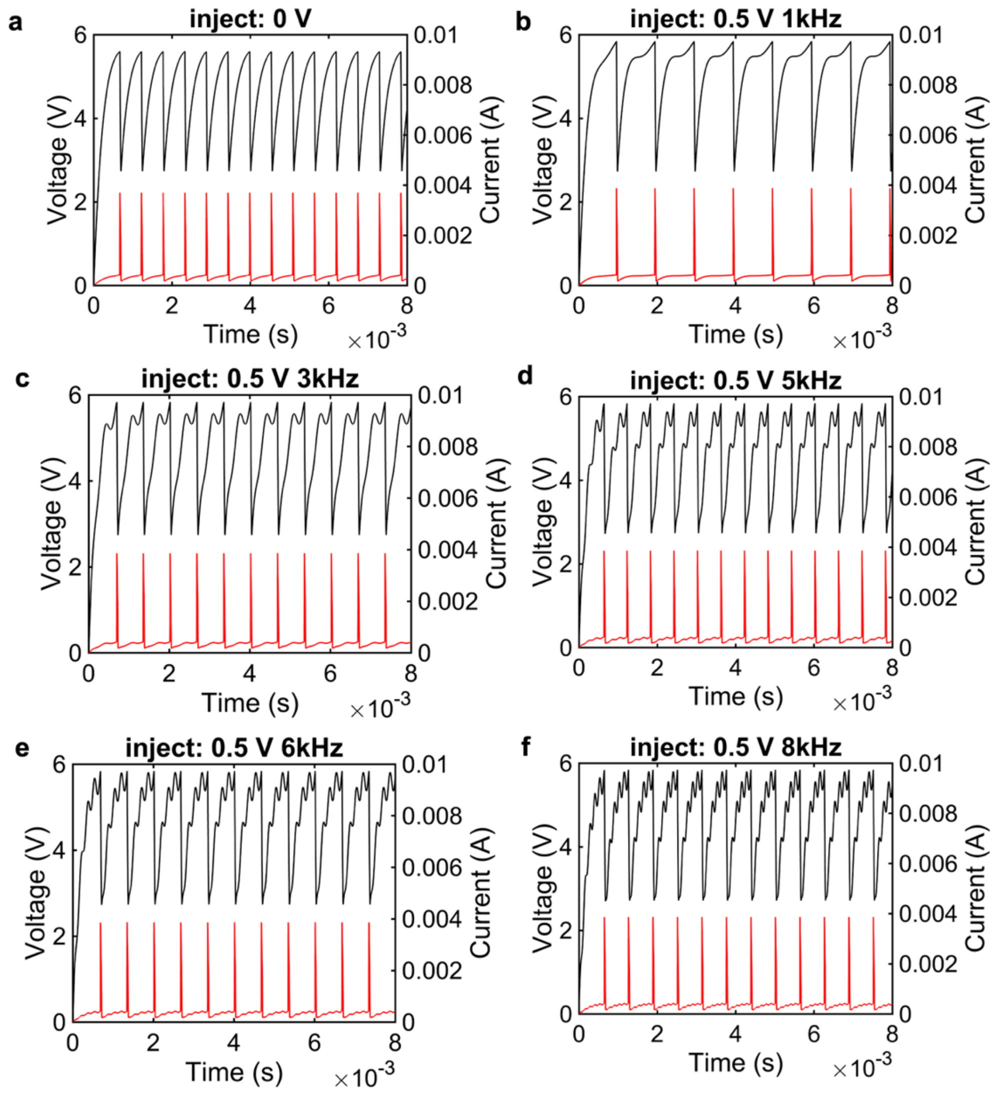

Supplementary Figure 18. The simulation results of the frequency modulation effect of VO_2 device biased on CEOC point (0.42 mA). The inject amplitude is 0.5 V. The inject capacitance is 15 nF. (a) inject signal = 0, the intrinsic frequency is 1.85 kHz. (b) the inject frequency is 1 kHz, the output frequency is locking to 1 kHz. (c) the inject frequency is 3 kHz, the output frequency=3 kHz/2=1.5 kHz. (d) the inject frequency is 5 kHz, the output frequency=5 kHz/3=1.67 kHz. (e) the inject frequency is 6 kHz, the output frequency=6 kHz/4=1.5 kHz. (f) the inject frequency is 8 kHz, the output frequency=8 kHz/5=1.6 kHz.

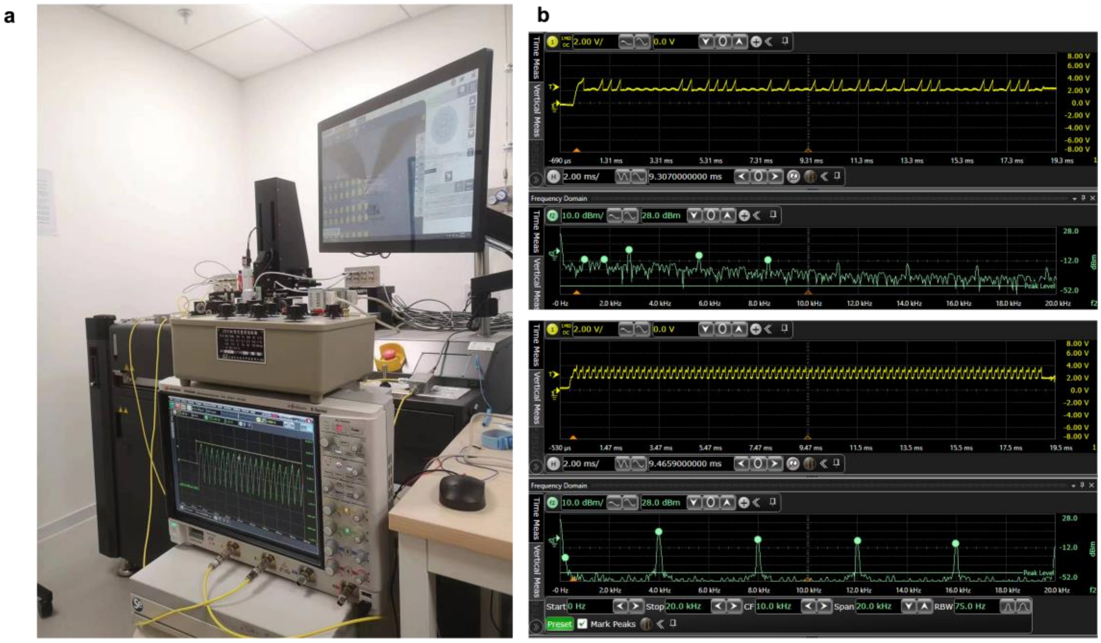

Supplementary Figure 19. Photos of experimental testing instruments and the panel display results of the oscilloscopes.

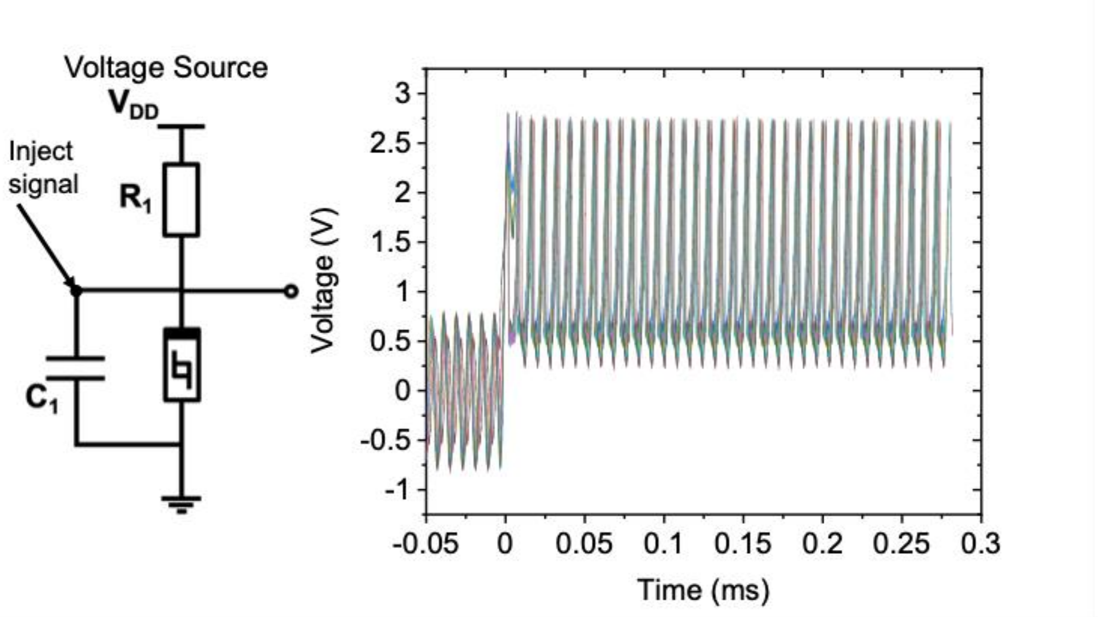

Supplementary Figure 20. The inject influence of voltage-source-based oscillators. With proper strength inject signals (1.5 V), the oscillators' frequency will be forced to be same to the inject signal's frequency. The amplitude of inject signal is 1.5 V. We first added the inject signal, then applied the voltage source (Otherwise, the oscilloscope will be triggered incorrectly). Therefore, when t<0, the voltage output is a sine oscillation. When t>0, the VO_2 device oscillated and was locked to the same frequency of the inject signal. Therefore, the voltage jump comes from the applied voltage source and shows the device's inject locking behaviour while oscillating.

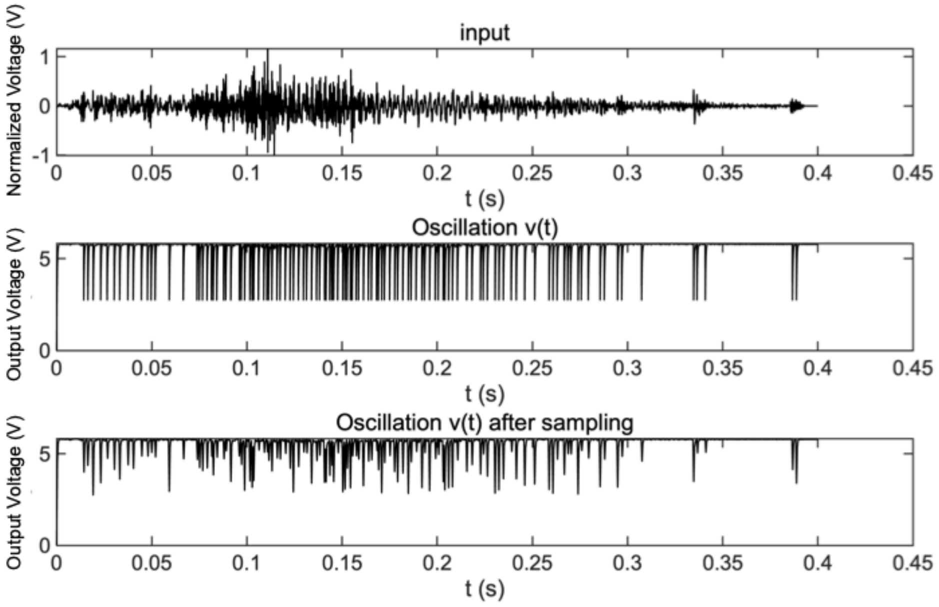

Supplementary Figure 21. The temporal information of the spoken digit 3 and the oscillation behaviour after CEOC oscillator's dynamics conversion and sampling for the neural network.

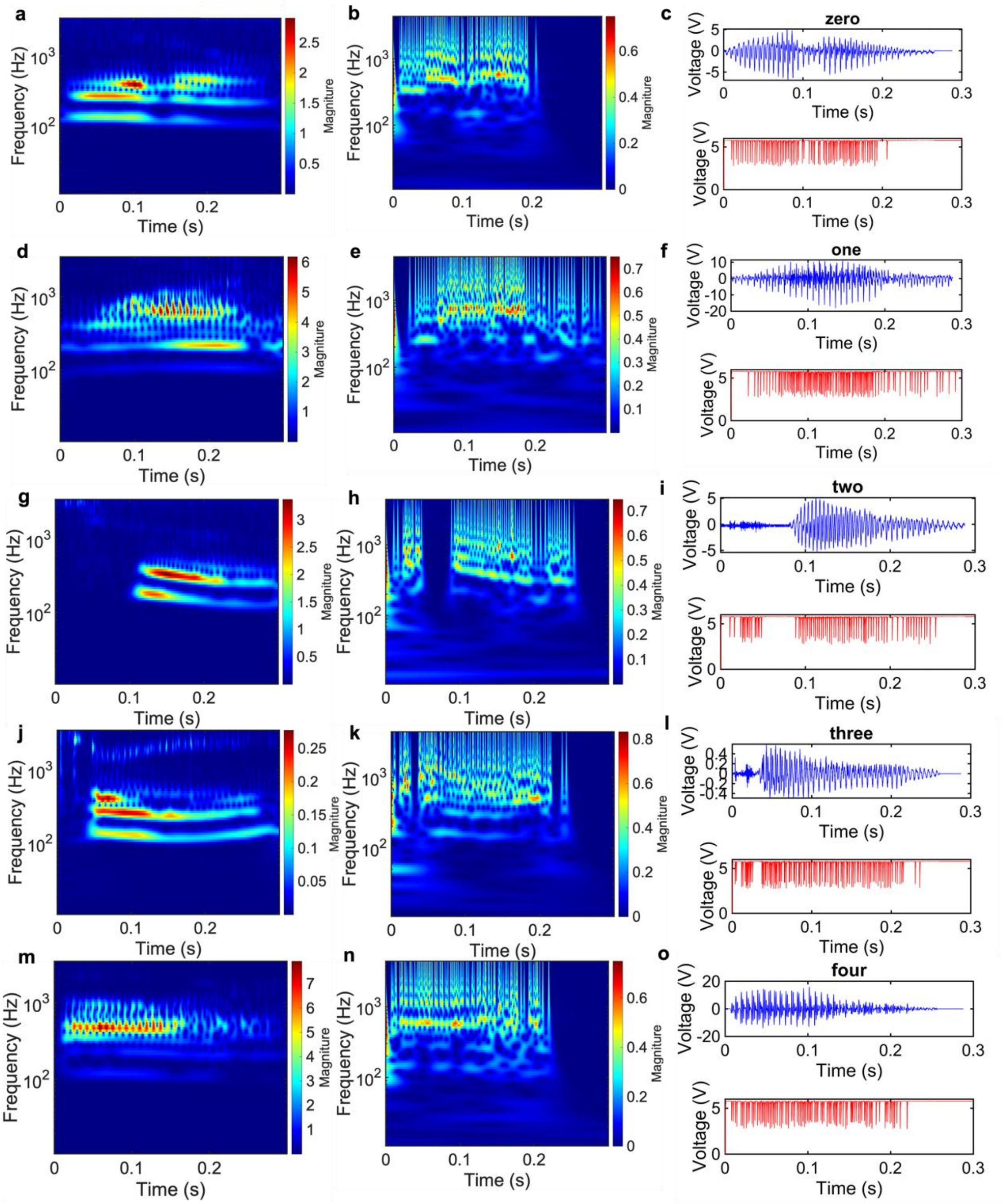

Supplementary Figure 22. The temporal information and wavelet transform of number 0-4 before and after CEOC oscillator's dynamics conversion. (a), (d), (g), (j), (m) show the wavelet transform of raw spoken digit data 0-4. (b), (e), (h), (k), (n) show the wavelet transform of CEOC oscillator's filtered digit data 0-4. (c), (f), (i), (l), (o)

show the comparison of these two signal waveforms in time domain of digit data 0-4. The upper is the raw spoken data and the below is the filtered data.

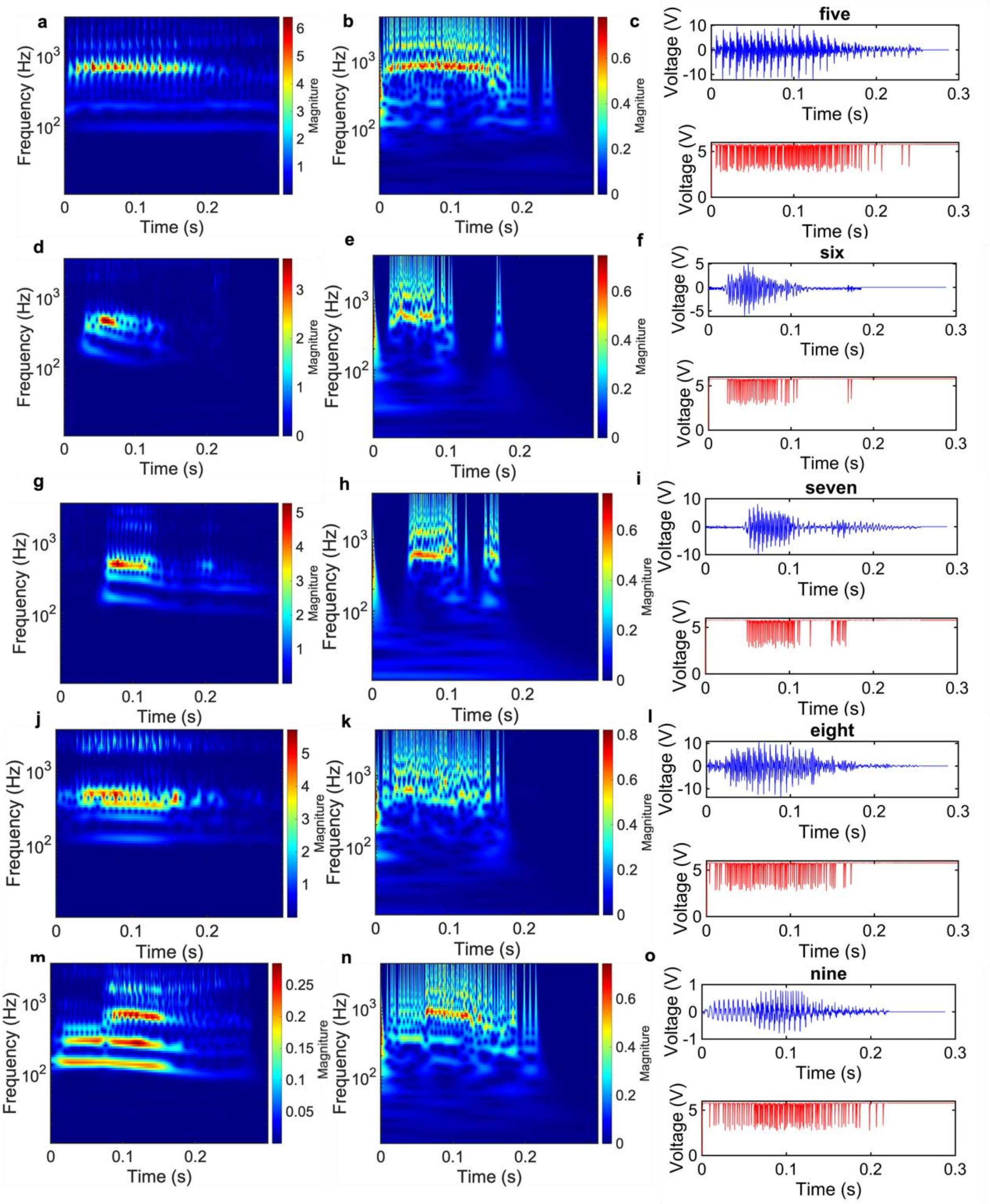

Supplementary Figure 23. The temporal information and wavelet transform of number 5-9 before and after CEOC oscillator's dynamics conversion. (a), (d), (g), (j), (m)

show the wavelet transform of raw spoken digit data 5-9. (b), (e), (h), (k), (n) show the wavelet transform of CEOC oscillator's filtered digit data 5-9. (c), (f), (i), (l), (o) show the comparison of these two signal waveforms in time domain of digit data 5-9. The upper is the raw spoken data and the below is the filtered data.

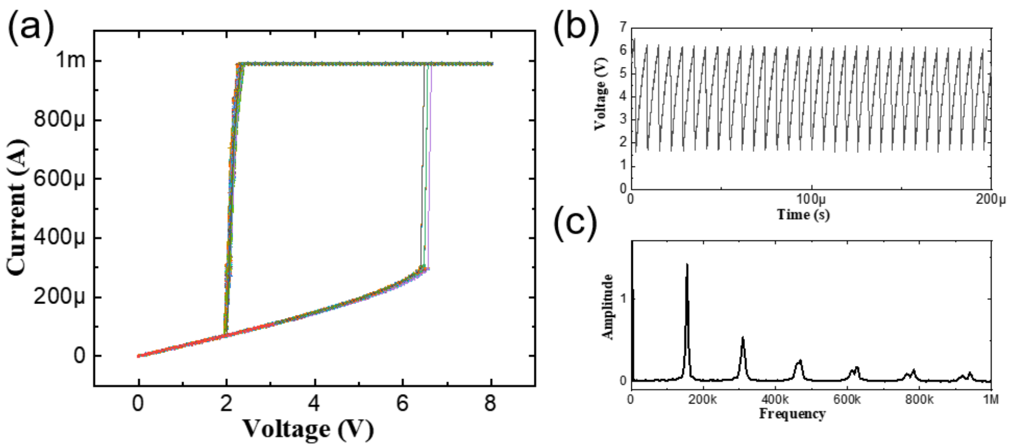

Supplementary Figure 24. (a) DC voltage sweeps of single VO_2 device over 300 cycles showing high consistency. (b) device oscillation characteristics and (c) corresponding Fourier transform results show the consistency of the device oscillation also show high consistency.

Supplementary Note.1 The principle of local activity

The local activity theory has profound connotations. Here, we give a brief introduction to this work to ensure that the work is aimed at a wider range of readers in various fields.

In memristor theory, the physical dynamics in nanodevices can be described using the inner state variables and the variables' nonlinear differential equations. When they exhibit a certain degree of nonlinearity, the devices will present a local active region, as shown in Supplementary Figure 25a. Active memristors that show current-controlled NDR (negative differential resistance), which is an "S"-shaped I–V trajectory in current sweeps, as shown in Supplementary Figure 25b. When the device is biased within the negative NDR region, local active memristors can generate AC signal gains greater than 1, act as amplifiers[32], or can excite oscillations in circuits with appropriate reactive elements[13-15,31]. These behaviours can be defined mathematically by local active principle.

Consider a physical device's nonlinear dynamics, which can be represented as:

$$\dot{\boldsymbol{x}} = f(\boldsymbol{x}) \tag{1}$$

Where $\boldsymbol{x}$ represents the state variable. For an equilibrium point $f(\boldsymbol{x}^{*}) = 0$, The local stable can be defined as:

$$\lim_{t\to\infty} \boldsymbol{x}(t) = \boldsymbol{x}^*$$

(2)

which means device can be biased at a stable state under a direct-current (DC) source and small disturbances will not cause it to deviate stable state. Define *di(t)* as the fluctuation of the current. The fluctuation *di(t)* causes the device's inner state variable x(t) change *dx(t),* which in turn triggers the device's voltage *v(t)* change *dv(t).* The local passive and local active can be defined as:

$$Local\ Passive \overset{\text{def}}{=} \int_0^t \delta i(t) \times \delta v(t) dt \geq 0 \tag{3}$$

$$Local\ Active \overset{\text{def}}{=} \int_0^t \delta i(t) \times \delta v(t) dt < 0 \tag{4}$$

Local passive region refers to the dissipative circuit elements. Local active region refers to the ability to amplify fluctuations while locally stable state means preventing small fluctuations from exploding without bounds.

The shift between local passive region and local active region will bring new computing advantages. Therefore, we bias the device at a critical current point, which we defined as the critical EOC (CEOC). This marks the transition from positive differential resistance to negative differential resistance (NDR). Near this transition point, small signal disturbance leads to new firing dynamics, which can be controlled by the signal’s frequency, as shown in Supplementary Figure 25b. We also found the value of the CEOC current is constant within a certain temperature range. The physical reason for this phenomenon is the electric-driven phase transition caused by an excessive carrier concentration due to devices' larger aspect ratios. Therefore, the total circuit diagram is shown in Supplementary Figure 25b, which shows the

oscillators can transform the expression of a chaotic temporal signal in phase space, preliminarily showing the ability to extract information from complex signals.

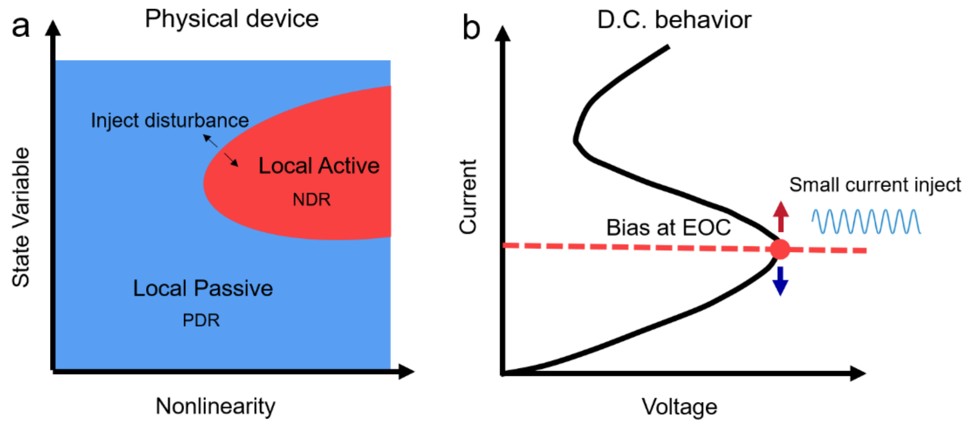

Supplementary Figure 25. a The nonlinearity of physical dynamics triggers the emergence of local active region. b Typical I-V curve of local active devices with the edge of chaos region featured as negative differential resistance. A small current injected on EOC alters its bias state, thereby inducing complex dynamics.

Supplementary Note 2. The parasitic capacitance from circuits

The local active memristor will be easily embedded in the measurement circuits. The parasitic capacitance cannot be neglected in testing. The parasitic capacitance from the planar VO_2 device itself and substrate is less than 1 pF (S is about 20nm*1um, d is 400 nm). The main parasitic capacitance is from current source of test instruments and the coaxial lines. When directly connecting VO_2 devices and proper fixed resistors in series (without external capacitance), the circuit can also oscillate under the proper voltage source. The parasitic capacitance affects the amplitude of the change in oscillator's intrinsic frequency. Therefore, the parasitic capacitance can be estimate from the testing results as around 1 nF. In the following model simulation, we keep using the 1 nF Ceff. This big parallel parasitic capacitance will affect dynamic device testing under the current sweep.

Besides, the probes from the oscilloscope will also bring additional capacitance. The passive probes have relatively high parasitic capacitance and low impedance when the oscilloscope has 1M ohm internal resistance. The active probes have relatively low parasitic capacitance and high impedance when the oscilloscope has 50 ohm internal resistance. To reduce the influence of the external circuits, we also use active probes to measure the quasi-static I-V sweep curve and oscillation, as shown in Supplementary Fig. 4-5. The active probe can decrease the capacitance influence of the measurement circuits. Here the active probe we used consists of amplifier

Keysight MX0025A and probe head MX0100A. It has 25 kohm / 0.17 pF single-ended input impedance. Therefore, this type of active probe will introduce additional parallel resistors (25 kohm) in our experiments. As shown in Supplementary Fig. 4, the resistance of point A is 10710 ohm and point B is 7460 ohm. Considering the 25kohm input impedance from the active probes, 10710 ohm // 25 kohm = 7498 ohm. The results mean that the difference in the IV curve from different probes is due to this parallel resistor, and the single VO_2 device's dynamic behaviours are actually the same with both passive probes and active probes. It is crucial to eliminate potential interference from measurement circuits for testing local active memristors. Therefore, in the following measurements, we can just use passive probes, because it will not change the behaviours of local active oscillators

Supplementary Note 3. The details of the compact model

As shown in Fig. 3a, the processes of VO_2 scanning are analyzed and incorporated into a unified physical modelling framework, with 8 dynamic processes outlined as follows:

0-1: Positive differential resistance& local passive: it's expressed as a linear change in conductivity with temperature.

1-2: Sharp negative differential resistance: At approximately 58 ℃, it is expressed as vanadium oxide beginning a phase transition, creating a thermal phase transition channel at the center of the device. At this point, there is an abnormal decrease in the

IV product, while the temperature remains continuous, indicating that the cooling law must include an additional external constant term. This constant term, represented as *C*, can be explained as the volume enthalpy of phase transition during thermal conduction.

2-3: Negative differential resistance & local active: Approximately occurring between 58 ℃ and 65 ℃, it is expressed as a nonlinear synchronous change in electrical and thermal conductivity during the vanadium oxide phase transition. The key point is that if only the nonlinearity of conductivity is considered at this time, it is impossible to fit parameters that conform to physical knowledge, because the temperature difference cannot express the significant changes in the IV product. We adopted the erf error function to fit G_{th} and G_{el} separately. At this point, both G_{th} and G_{el} increase monotonically.

$$R = (erfc\left(\frac{(T-T_1)}{a}\right) \times \left(1-\frac{T}{T_c}\right) \times R_H + R_L \quad (7)$$

$$G_{th} = \mathrm{erf}\left(\frac{T-T_2}{b}\right) \times G_{th0} + G_{th1} \quad (8)$$

Where T_1, T_2, T_c, a, and b are constants based on phase change material characteristics. R_H means high resistance state, and R_L means low resistance state. The erf error function may appear complex, but it conforms to the phase transition law and can minimize the number of physical fitting variables required. Other functions, such as sigmoid, can also realize similar effects, but the fitting accuracy will decrease, and it may require compensation terms.

3-5: At this time, the current continues to change, but the temperature suddenly rises due to the rapid decline in the device’s thermal conductivity, forming hysteresis. This

can be understood as a sudden contraction of the phase transition area in the device. During the steady-state temperature scanning process, a continuous temperature range of 3-4-5 should be observed. During the 5-6 IV flyback process, the device temperature decreases. The reason it does not immediately return to the initial state is due to the hysteresis in the temperature scanning of vanadium oxide, which also exhibits asymmetric positive-negative memory characteristics. This is expressed here as hysteresis in thermal conductivity.

4-6: The temperature continues to decrease to around 60 ℃, at which point a transition occurs and the device jumps back to the continuous NDR region of 8.

Supplementary Note 4: The detailed parameters of the CNN.

First, the original audio signal is preprocessed through FFT to transform to a spectrum (40, 50) for extracting MFCC features.

The CNN structure is:

```
model = Sequential([

Conv2D(32, kernel_size=(3, 3), activation='relu', input_shape=(40, 50, 1)),

MaxPooling2D(pool_size=(2, 2)),

Dropout(0.3),

Conv2D(64, kernel_size=(3, 3), activation='relu'),

MaxPooling2D(pool_size=(2, 2)),

Dropout(0.3),
```

```
Flatten(),

Dense(128, activation='relu'),

Dropout(0.3),

Dense(10, activation='softmax')    # 10 classes for digits 0-9)
```

Therefore, the total multiplication operation in Conv Layer is:

3*3*38*48*32+2*2*17*22*64 = 621056

Supplementary Note 5: Energy consumption estimation

The energy consumption is mainly associated with the fire and reset processes. The oscillator circuit is shown in Supplementary Fig. 5**:**

When we measure the output voltage V_{out}, the transient power consumption can be computed by:

$P = V_{out} * I_{bias}$

Then the total energy consumption over a time period T can be calculated as:

$$E = \int_0^T I_{bias} \times V_{out}(t) dt$$

Therefore, the energy per spike can be calculated as:

$E_{spike} = Energy / frequency$

Using experimental data from the oscillations, the oscillator's energy consumption can be computed in this method. It is 1.76-1.94 mW.

Supplementary Note 6: Energy Comparison

To have a fair comparison, we chose the same spoken digit classification task respectively on CPU, FPGA, reservoir computing and this work.

The comparison only concludes the reservoir part.

For CPU, we chose Intel(R) Core(TM) i5-4460 CPU @ 3.20GHz as the CPU. According to M. L. Aloma et al [S1]. Its power consumption is 13.7 W and the time step is 4us.

For FPGA, Aloma et al. also estimated the power to be 83 mW, and the process time needed for a single input to be 1.734μs.

The above was also described in detail in John, et al[S2].

For reservoir computing based on volatile memristors, we chose two representative work from Moon, et al[S2] and Zhong, et al[S3]

Moon, et al use WO_x memristors, They use the NIST TI46 database, preprocessed using Lyon's passive ear model based on human cochlear channels. The preprocessing transforms the sound waveforms into a set of 50-dimensional vectors (corresponding to the frequency channels) with up to 40 time steps. The power consumption for processing a single input is approximately 3.0 V × 100 μA = 300 μW. Therefore, for the total reservoir, the power is 300 uW×50 = 15mW. The write pulse width is 10 μs.

Moon, et al use WO_x memristors, They use the NIST TI46 database, preprocessed using Lyon's passive ear model based on human cochlear channels. The preprocessing transforms the sound waveforms into a set of 50-dimensional vectors (corresponding

to the frequency channels) with up to 40 time steps. The power consumption for processing a single input is approximately 3.0 V × 100 μA = 300 μW. Therefore, for the total reservoir (50 devices), the power is 300 uW×50 = 15mW. The single write pulse width is 10 μs.

Zhong, et al use TiO_x/TaO_x memristors. They use the NIST TI46 database, preprocessed using Lyon's passive ear model based on human cochlear channels. Besides, they add a mask to further pre-processing. In each interval of duration τ, the spectrum signal is multiplied by a $64 \times M$ mask matrix to generate the input voltage sequence with a time step δ equal to $1/M$ of τ, where M is the mask length. The power consumption for processing a single input is approximately 50 μW. Therefore, for the total reservoir (64 devices), the power ist 50 uW×64 = 3.2 mW. The pulse width used in the current system is 120 μs.

Supplementary Note 7: Comparison with other physical computing methods

We compare this work with Momeni, et al [S4] and Wright, et al [S5]. They both use optical diffraction and acoustic oscillators to realize the nonlinear processing for audio signals. These devices' order is >=2. The training method they used is physical backpropagation and Forward-Forward Learning. Their network structure is Physical layers + FC + physcial layer + FC+⋯. However, due to they both use large size devices rather than integrated devices, the area efficiency and energy efficiency are far less than our method based on VO_2 devices.

Supplementary Note 8: Discussion of amplitude and frequency of injected signals

We chose the audio signal for application because its frequency domain has the most important information. The classical classification method always applied a short-term Fourier transform to make a time-domain to frequency-domain conversion for feature extraction. The physical dynamic in VO_2 will have a frequency selectivity and tunable nonlinearity. As we can see, the large amplitude or higher frequency will both induce the firing of the VO_2 oscillator at the CEOC point, but their behaviour is different. The higher frequency part will cause the accumulation and jitter on membrane potential while the large amplitude will only cause a rapid increase on membrane potential. Besides, different frequency will cause different firing behaviours as shown in Figure 4, while different amplitude will only cause the different firing frequencies. If we chose the inject capacitance right, the Intrinsic oscillation frequency of VO_2 is similar to the audio signal. At that time, the VO_2 oscillator has the strongest frequency selectivity to the audio signal. Therefore, although the amplitude and frequency of the speech data cannot be decoupled easily in oscillator computing from the formula, the different influence of them will help classification.

Supplementary Table.1 Comparison with physical computing methods

	**Physical neural network**[S4, S5]	**EOC oscillator computing (this work)**
**Device order**	>=2	>=3
**Computational Source**	Nonlinear oscillator dynamic	Edge of chaos oscillator dynamic
**Device number**	1*N	1
**Feature extraction**	Relying on physical device's nonlinear dynamic	Relying on device's edge of chaos dynamic
**Network Structure**	Physical layers + FC + physcial layer + FC …	1 device + 2 layer FC
**Power**	\	1.76-1.94 mW

Supplementary Table 2. The compact model parameters

Tamb (Environmental temperature）	30
T_1（Thermal conductivity transition temperature）	65
T_2（Lower limit temperature of hysteresis）	60
T_3（Conductivity transition temperature）	58
C_{th}	1e-10
Cap	1e-9
R_0	1.663e4
R_H	1.6e4
R_L	1.5e3
alpha	0.0075
beta	100
C	0.000415
T_{el}	62
T_{th1}	70
T_{th2}	72
delta_T_{el}	10
delta_T_{th}	8
Gth_0	8.5e-5
Gth_1	10e-5

Gth_2	5e-5
delta_Gth	10e-5

f_{el} (T) = (erfc((T-T_{el})/delta_T_{el}))*((1-T/beta))*RH +RL;

$f1_{th}$ (T) = erf((T-T_{th1})/delta_T_{th})*delta_Gth+delta_Gth+Gth_0;

$f2_{th}$ (T) =erf((T-T_{th2})/ delta_T_{th})*Gth_2 +Gth_1;

Supplementary Reference:

[S1] Alomar ML, et al. Digital implementation of a single dynamical node reservoir computer. *IEEE Trans Circuits Syst II* 2015; **62**: 977–81.

[S2] Moon J, Ma W, Shin JH, et al. Temporal data classification and forecasting using a memristor-based reservoir computing system. *Nat Electron* 2019; **2**: 480–87.

[S3] Zhong Y, Tang J, Li X, et al. Dynamic memristor-based reservoir computing for high-efficiency temporal signal processing. *Nat Commun* 2021; **12**: 408.

[S4] Momeni A, Rahmani B, Malléjac M, et al. Backpropagation-free training of deep physical neural networks. *Science* 2023; **382**: 1297–303.

[S5] Wright LG, Onodera T, Stein MM, et al. Deep physical neural networks trained with backpropagation. *Nature* 2022; **601**: 549–55.
